# NMDARs Drive the Expression of Neuropsychiatric Disorder Risk Genes Within GABAergic Interneuron Subtypes in the Juvenile Brain

**DOI:** 10.3389/fnmol.2021.712609

**Published:** 2021-09-14

**Authors:** Vivek Mahadevan, Apratim Mitra, Yajun Zhang, Xiaoqing Yuan, Areg Peltekian, Ramesh Chittajallu, Caroline Esnault, Dragan Maric, Christopher Rhodes, Kenneth A. Pelkey, Ryan Dale, Timothy J. Petros, Chris J. McBain

**Affiliations:** ^1^Section on Cellular and Synaptic Physiology, Eunice Kennedy Shriver National Institute of Child Health and Human Development (NICHD), Bethesda, MD, United States; ^2^Bioinformatics and Scientific Programming Core, NICHD, Bethesda, MD, United States; ^3^Unit on Cellular and Molecular Neurodevelopment, NICHD, Bethesda, MD, United States; ^4^Flow and Imaging Cytometry Core Facility, National Institute of Neurological Disorders and Stroke (NINDS), Bethesda, MD, United States

**Keywords:** GABAergic interneurons, medial ganglionic eminence, transcriptional regulation, neurodevelopmental disorders, schizophrenia, NMDAR-hypofunction, scRNAseq, Ribotag-seq

## Abstract

Medial ganglionic eminence (MGE)-derived parvalbumin (PV)+, somatostatin (SST)+and Neurogliaform (NGFC)-type cortical and hippocampal interneurons, have distinct molecular, anatomical, and physiological properties. However, the molecular mechanisms regulating their maturation remain poorly understood. Here, via single-cell transcriptomics, we show that the obligate NMDA-type glutamate receptor (NMDAR) subunit gene *Grin1* mediates transcriptional regulation of gene expression in specific subtypes of MGE-derived interneurons, leading to altered subtype abundances. Notably, MGE-specific early developmental Grin1 loss results in a broad downregulation of diverse transcriptional, synaptogenic and membrane excitability regulatory programs in the juvenile brain. These widespread gene expression abnormalities mirror aberrations that are typically associated with neurodevelopmental disorders. Our study hence provides a road map for the systematic examination of NMDAR signaling in interneuron subtypes, revealing potential MGE-specific genetic targets that could instruct future therapies of psychiatric disorders.

## Introduction

Medial ganglionic eminence (MGE)-derived forebrain GABAergic interneurons comprise the parvalbumin- containing (PV) and somatostatin-containing (SST) subpopulations throughout the entire forebrain accounting for approximately 60% of all cortical interneurons (Pelkey et al., 2017; Wamsley and Fishell, 2017). In addition, approximately half of all hippocampal neurogliaform-type cells (NGFCs), including the Ivy cells, originate from the MGE (Tricoire et al., 2010, 2011). Interestingly, though only rarely found in rodent neocortex, such MGE-derived NGFCs are significantly more populous in primate and human neocortex (Krienen et al., 2020). While PV neurons exert robust somatic inhibition, the SST and NGFCs mediate domain-specific dendritic inhibition on their downstream pyramidal neuron targets (Pelkey et al., 2017; Wamsley and Fishell, 2017). Collectively, the interneurons shape diverse aspects of cortical and hippocampal circuit maturation and regulate information processing in mature circuits by maintaining appropriate excitation-inhibition (E-I) balance. Interneuron-specific impairments are increasingly considered central to the etiology of a number of neural circuit disorders because, numerous human CNS-disorder risk-genes are expressed in a manner specific to the development of GABAergic interneurons (Marín, 2012). Hence, there is a critical need to examine the molecular mechanisms that regulate the development and maturation of GABAergic interneurons.

Immature interneurons express different glutamate receptor subunits including the NMDA-type iGluR (NMDAR) and AMPA/Kainate-type iGluR (AMPAR/KAR) (Monyer et al., 1994; Soria, 2002; Manent, 2006), prior to the expression of any functional synapses. Multiple evidence indicates a critical role for neuronal activity, particularly through ionotropic glutamate receptors (iGluRs), in driving the development of MGE-derived interneurons (Matta et al., 2013; De Marco García et al., 2015; Pelkey et al., 2017; Priya et al., 2018; Zoodsma et al., 2020). But unlike the mature interneurons, where iGluRs are established to mediate synaptic transmission and plasticity, the precise roles for the iGluR subunits within developing interneurons are only emerging. Since the developing brain contains higher ambient glutamate than the adult brain (Hanson et al., 2019), the interneuron-expressed-iGluRs are thought to mediate trophic signaling while regulating the migration, survival, morphological and physiological maturation of interneuron development (Manent, 2006; Yozu et al., 2007; Bortone and Polleux, 2009; Desfeux et al., 2010; De Marco García et al., 2011, 2015; Kelsch et al., 2012; Le Magueresse and Monyer, 2013; Chittajallu et al., 2017; Hanson et al., 2019; Akgül and McBain, 2020).

An impairment in NMDAR signaling within GABAergic interneurons is emerging to be a key driver of juvenile-onset neural circuit disorders (Belforte et al., 2010; Rico and Marín, 2011; Nakazawa et al., 2017; Nakazawa and Sapkota, 2020). Particularly, early postnatal ablation of the obligate NMDAR subunit gene *Grin1* in GABAergic interneurons (Belforte et al., 2010; Nakao et al., 2018; Bygrave et al., 2019; Alvarez et al., 2020) resembles global *Grin1*-loss in their constellation of schizophrenia-like behavioral and neural circuit aberrations (Mohn et al., 1999). But an adult-onset (Belforte et al., 2010), or PV-exclusive (Korotkova et al., 2010; Bygrave et al., 2016), or glutamatergic neuron-exclusive *Grin1* ablation (Tatard-Leitman et al., 2015), fails to recapitulate similar behavioral abnormalities in mice. This demonstrates that NMDAR signaling plays a crucial role in determining GABAergic interneuron development that later impacts on a variety of neural circuit properties in the juvenile brain. Despite the importance of developmental NMDAR function in interneurons and its relevance to human neural circuit disorders, a comprehensive interrogation of the impact of developmental NMDAR ablation in MGE-derived interneurons, particularly across the juvenile brain is currently lacking.

NMDARs are multi-functional molecular machines that uniquely couple glutamate-induced Ca^2+^ influx pathways with gene expression regulatory programs, referred to as excitation-transcription (E-T) coupling in mature circuits (Yap and Greenberg, 2018). However, it is not clear whether the NMDAR-mediated Ca^2+^ cascades engage the transcriptional programs necessary for development and maturation in developing MGE-derived interneurons (Komuro and Rakic, 1992; Soria, 2002; Bortone and Polleux, 2009). In the present study, we examine the impact of, (i) an early developmental loss of NMDAR function in GABAergic interneurons and, (ii) the juvenile-onset NMDAR-mediated E-T coupling within MGE-derived interneurons. In particular, we conditionally deleted *Grin1* in MGE progenitors that give rise to cortical and hippocampal PV, SST, and NGFC subsets, using the *Nkx2-1*-Cre mouse line (Xu et al., 2008; Tricoire et al., 2010, 2011). In this model, the *Nkx2-1*-driven Cre expression reported in proliferating interneuron progenitors, allows for examining of the impact of embryonic loss of *Grin1* activity across all subsets of MGE-derived interneurons. Applying unbiased single-cell RNA sequencing (scRNAseq), MGE-interneuron-specific Ribotag-seq, quantitative immunostaining and *in situ* RNAscope analyses in the juvenile-brain, we establish that NMDAR-mediated transcriptional cascades regulate MGE subtype abundances, and the expression of diverse transcriptional, synaptogenic and membrane excitability genetic programs. Notably, we identify several disease-relevant genes that are misexpressed in MGE-derived interneurons upon *Grin1*-ablation, providing a broad road map for examination of MGE-derived interneuron-subtype-specific regulation via NMDAR signaling.

## Results

### scRNAseq Recapitulates Cardinal MGE Subtypes and a Continuum of Molecular Profiles

**FIGURE 1:**
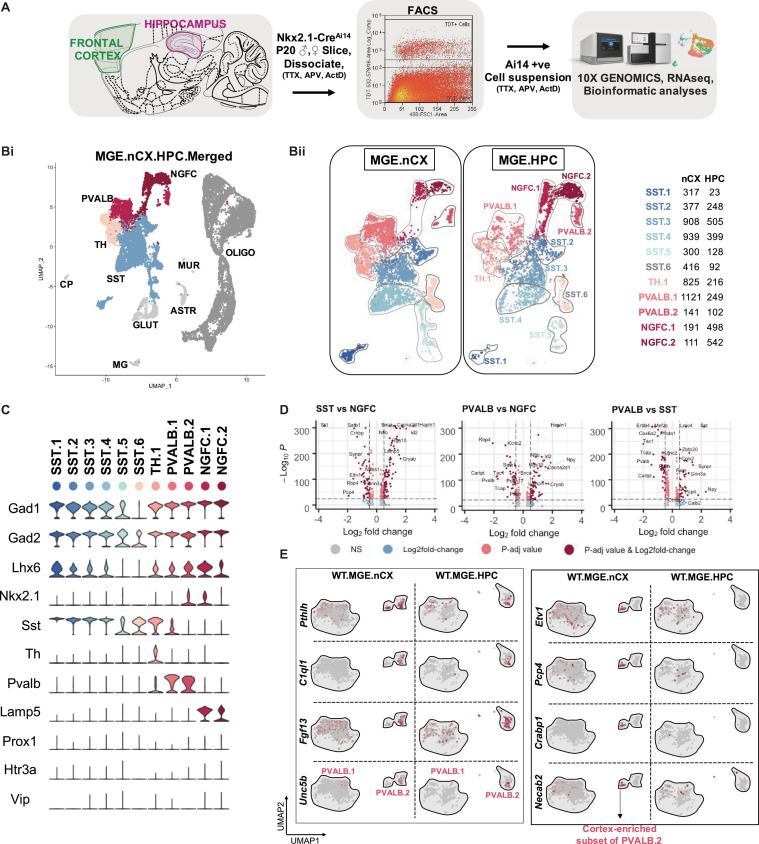
Identification of MGE-derived interneuron subtypes in the cortex and hippocampus. **(A)** Overview of the experimental workflow. **(Bi)** Uniform Manifold Approximation and Projection (UMAP) dimensional reduction of 19,028 single-cell transcriptomes (9,064 from frontal cortex and 9,964 from hippocampus of 6 mouse brains, 3 biological replicates), showing the cardinal MGE populations. Cell clusters were color coded and annotated *post hoc* based on their transcriptional profile identities (Cell type abbreviations: PVALB, Parvalbumin; NGFC, Neurogliaform; TH, Tyrosine Hydroxylase; SST, Somatostatin; GLUT, Glutamatergic; CP, Choroid Plexus; MG, Microglia; ASTR, Astrocyte; MUR, Mural; OLIGO, Oligodendrocyte). **(Bii)** UMAP visualization of 11 MGE-derived interneuron subtypes from neocortex (MGE.nCX) and hippocampus (MGE.HPC), and the recovery of cell numbers from the subtypes. **(Biii)** Table indicating the number of *Gad1/Gad2* + cells recovered in each MGE subtype from the neocortex and hippocampus, and the defining genes enriched in each subtype. **(C)** Violin plot showing the distribution of expression levels of well-known representative cell-type-enriched marker genes across the11 MGE subtypes. **(D)** –log10 False Discovery Rate (FDR) vs. log2 fold change (FC) between each of the MGE cardinal class, representing the top enriched markers at a fold change ≥ 0.5 and FDR < 10e-25. **(E)** UMAP representation of PVALB clusters highlighting the cortex-specific enrichment of *Pthlh*-expressing PVALB.2 subtype that is not observed in the hippocampus.

To examine the molecular heterogeneity of MGE-derived GABAergic interneurons by scRNAseq, we microdissected frontal cortex/neocortex (nCX) and hippocampus (HPC) from fresh brain slices obtained from PD18-20 *Nkx2.1*-*Cre:Ai14* mouse (Figure 1A and Figure 1-Supplement 1A). Ai14-TdTomato (TdT^+^) single-cell suspensions were harvested by fluorescence-activated cell sorting (FACS) using stringent gating constraints including viability and doublet discrimination (Figure 1-Supplement 1B) as previously described (Tanaka et al., 2008; Harris et al., 2018; Muñoz-Manchado et al., 2018), and subsequently processed through the 10X Genomics Chromium controller. 9064 and 9964 TdT^+^ cells were recovered from cortex and hippocampus, respectively across 3 biological replicates. To minimize the effect of excitotoxicity and stress-related transcriptional noise, the tissue processing, FACS, and sample collection steps were performed in buffers supplemented with Tetrodotoxin (TTX), DL -2-Amino-5-phosphonopentanoic acid (APV) and Actinomycin-D (Act-D) (Wu Y. E. et al., 2017). Because we observed concordant cell clustering across the replicates during preliminary analysis by Seurat v3 (Butler et al., 2018; Stuart et al., 2019; Figure 1-Supplement 2A), the replicates were pooled for in-depth analysis. Subsequent clustering and marker gene analyses revealed that ∼62 and 33% of the TdT^+^ MGE-sorts from cortex and hippocampus, respectively, express classical GABA markers including *Gad1*, *Gad2*, *Lhx6*; and the MGE-subclass markers *Pvalb*, *Sst*, and *Lamp5*, marking PV and SST, NGFC subsets, respectively (Figure 1B and Figure 1-Supplement 3A). While we did not recover cells expressing the CGE-markers *Prox1*, *Htr3a* or *Vip*, we recovered a minor fraction of cells corresponding to glutamatergic neurons, astrocytes and microglia. In addition, ∼25 and 71% TdT^+^ MGE-sorts from cortex and hippocampus, respectively were enriched in oligodendrocytes marked by *Olig1* expression across all replicates (Figure 1-Supplements 2B,C). However, we focused our subsequent analyses on the 5656 and 3002 *Gad1*/*Gad2* positive cortical and hippocampal MGE-derived interneurons.

Unbiased cell clustering by Seurat v3 identified six subtypes of SST, two subtypes of PV, two subtypes of NGFCs, and one subtype of Tyrosine hydroxylase (TH) expressing interneurons, expressing the markers *Sst*, *Pvalb*, *Lamp5*, and *Th*, respectively, across the two brain regions examined (Figure 1C). Notably, all but two subtypes (SST.5 and SST.6) expressed high levels of *Lhx6*, and 2 clusters corresponding to PV.2 and NGFC.1 expressed *Nkx2.1* at this developmental time. While the PV- SST- and NGFC- clusters clearly exhibited robust gene expression differences among each other (Figure 1D), the TH cluster appeared to express genes that correspond to both PV: SST clusters, including *Sst* and *Pvalb* expression (Figure 1C and Figure 1-Supplement 3B). Particularly, at this developmental window we could not observe robustly different gene expression variances between the cortical and hippocampal counterparts, barring a few marginal, but significant differences (Figure 1-Supplement 4B). This gave us sufficient rationale to perform subsequent analyses using the MGE-derived interneurons pooled from cortex and hippocampus.

Among the SST sub clusters, SST.1–5 uniquely expresses *Chodl*, *Igf2bp3*, *Cdh7*, *Pld5*, and *Nfix*, respectively, while SST.6 expresses only markers that are common with other SST clusters (Figure 1-Supplement 3B). With the exception of SST.6 the remaining SST-expressing subclusters are described in previous scRNAseq assays (Tasic et al., 2016; Paul et al., 2017; Figure 1-Supplement 5A). For example, the *Chodl*-expressing SST.1 cluster co-expresses high *Nos1*, *Tacr1*, *Penk*, and *Npy*, and it has been previously described as putative GABAergic long-range projection neurons. Clusters SST.2/3/4 express *Elfn1*, *Reln*, and *Grm1* characteristic of putative cortical martinotti and their hippocampal counterpart, oriens-lacunosum/moleculare (O-LM) cells (Tasic et al., 2016; Harris et al., 2018; Winterer et al., 2019; Figure 1-Supplement 5B). Lastly, *Zbtb20*-expressing SST.5 is predicted to be septal-projecting interneurons (Harris et al., 2018). Among the PV sub clusters, while both PVALB.1&2 coexpresses several common markers including *Pvalb*, *Kcnip2*, *Tcap*, and *Kcnc1* there are several notable differences between the two clusters. PVALB.1 appears to contain continuous, but non-overlapping populations expressing *Syt2* representing putative fast-spiking basket cells or *Rbp4/Sst* containing putative bistratified cells (Pelkey et al., 2017; Harris et al., 2018; Tasic et al., 2018; Figure 1-Supplement 5D). PVALB.2 contains cells that uniquely expresses *Pthlh*, *C1ql1*, *Fgf13*, and *Unc5b* representing putative axo-axonic chandelier cells (Paul et al., 2017; Harris et al., 2018; Favuzzi et al., 2019). We also observed a TH cluster, which, in addition to expressing several genes common to the SST: PV clusters, expresses several unique genes including *Rasgrp1*, *Bcl6*, *Myo1b* that segregated into mutually exclusive cluster space expressing *Crh* or *Nr4a2* (Figure 1-Supplements 3B, 5B). This cluster is also described previously as putative bistratified-like cells (Harris et al., 2018; Tasic et al., 2018). Among the NGFC sub clusters, while both NGFC.1&2 coexpress several common markers including *Lamp5*, *Hapln1*, *Cacna2d1*, *Sema3c*, and *Id2*, the NGFC.1 cluster uniquely expresses several genes like *Reln*, *Ngf*, *Egfr*, *Gabra5* that are not expressed by NGFC.2 (Figure 1-Supplement 3B). While the *Reln*-positive population represents MGE-derived neurogliaforms, the *Reln*-negative population may represent putative ivy cells (Harris et al., 2018; Figure 1-Supplement 5C). While the majority of the UMAP space aligns well between the cortical and hippocampal MGE-derived interneurons, we observed some regional differences as well (Figure 1-Supplements 3Ai,iii).

(i) First, we observed an increase in the HPC-expressed NGFC.1&2 in comparison to their cortical counterparts, consistent with preferential localization of MGE-derived NGFCs to HPC over nCX in rodents (Tricoire et al., 2011; Pelkey et al., 2017; Krienen et al., 2020). (ii) Next, the *Pthlh*-expressing PVALB.2 subcluster splits into two islands, only in the cortex and distinctly lacking from the hippocampus. Only one of the PVALB.2 islands expresses *C1ql1*, while the other cortex-enriched island expresses unique markers *Etv1*, *Cnr1*, *Pcp4*, *Crabp1*, *Necab2*, *Epha4*, and *Hapln1* (Figure 1E and Figure 1-Supplement 5D). Whether this represents a novel subclass of chandelier cells remains to be determined. (iii) Lastly, we also observed a distinction in the hippocampal SST.3 corresponding to a subset of O-LM interneurons (Figure 1-Supplement 3Aii). The overall MGE cell numbers indicate that the SST cells account for the majority of MGE cell population recovered in the scRNAseq assay from both brain regions (Figure 1-Supplements 3Aii,iii). The PV and TH clusters accounted for a greater share of MGE-derived interneurons in the nCX than in the HPC. While it is plausible these relative cell proportions may be skewed by differential survivability of these subtypes during tissue dissociation, sorting and single-cell barcoding, these relative percentages were similar across biological replicates.

### NMDAR Signaling Maintains MGE-Derived Interneuron Subtype Abundance

Because neuronal activity and glutamatergic signaling are known to regulate multiple facets of interneuronal development (De Marco García et al., 2011, 2015; Wamsley and Fishell, 2017; Denaxa et al., 2018; Priya et al., 2018; Wong et al., 2018), we hypothesized that the key obligate subunit *Grin1* and the NMDAR signaling complex may play an instructive role in determining MGE subtype identities. To test whether NMDAR signaling impacts the development and function of MGE-derived interneurons, we ablated them in MGE progenitors by crossing floxed-*Grin1* mice with the *Nkx2.1^*Cre*^* mouse line (Xu et al., 2008; Figure 1-Supplement 1A). The earliest expressions of *Nkx2.1* and *Grin1* in the developing rodent brains is reported around ∼embryonic day (ED) 10.5 and ∼ED14, respectively (Laurie and Seeburg, 1994; Monyer et al., 1994; Butt et al., 2008). Moreover, NMDAR-mediated Ca^2+^ signaling in migrating interneurons is reported by ∼ED16 (Soria, 2002). Because the expression and activity of *Nkx2.1* precedes *Grin1* expression, we rationalized that utilizing *Nkx2.1^*Cre*^* mouse will ablate *Grin1* and NMDAR signaling in MGE progenitors from the earliest developmental point. We sorted TdT^+^ cells from the cortex and hippocampus of *Nkx2.1^*Cre*^: Grin1^*fl/fl:Ai*14^* mice and performed scRNAseq using the 10X platform. The scRNAseq experiments were performed using juvenile mice (PD18–20) of both sexes and from the same litters as the wildtypes (WT) to enable subsequent direct comparison. Similar to the WT-datasets, the MGE-*Grin1^*fl/fl*^* mutants also revealed an enrichment of TdT + oligodendrocytes (Figure 2-Supplement 4B), however, we again focused our attention on the *Gad1/Gad2* positive interneurons.

**FIGURE 2:**
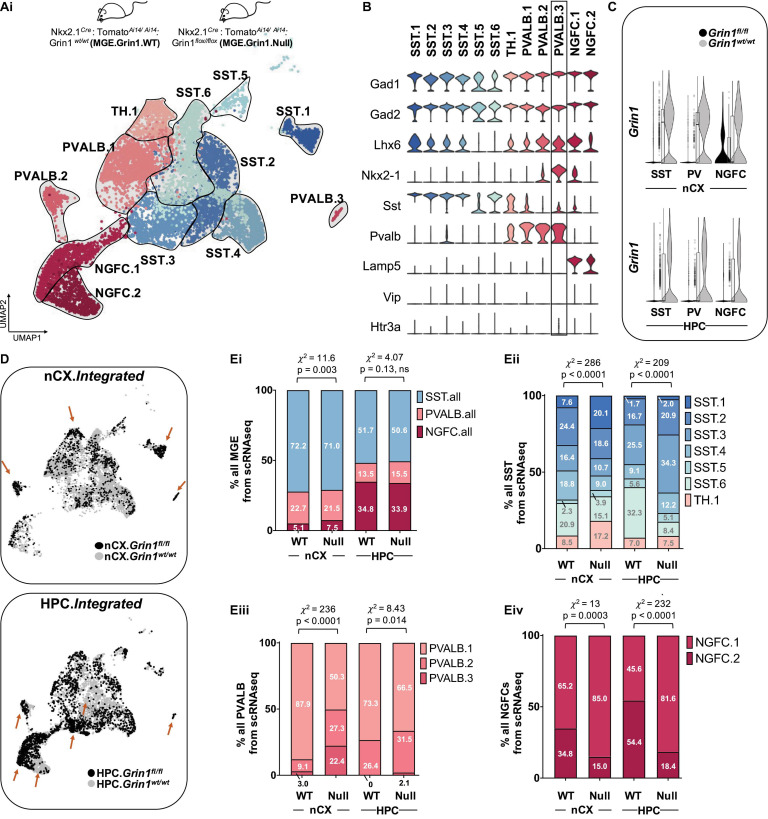
Altered interneuron subtype proportions upon *Grin1*-ablation. **(A)** Integrated UMAP visualization of 12 subtypes of MGE-derived interneurons obtained from neocortex (nCX) and hippocampus (HPC) of *Grin1^*wt/wt*^* and *Grin1^*fl/fl*^* mice. UMAP represents the following numbers of MGE-derived interneurons from *Grin1^*wt/wt*^* and *Grin1^*fl/fl*^*, respectively: 5624 (nCX.WT) and 1387 (nCX.NULL); 2998 (HPC.WT) and 2309 (HPC.NULL). 3 and 2 independent biological replicates of the scRNAseq assay from *Grin1^*wt/wt*^* and *Grin1^*fl/fl*^*, respectively. **(B)** Violin plot showing the distribution of expression levels of well-known representative cell-type-enriched marker genes across the 12 interneuron subtypes. **(C)** Violin plot from both genotypes indicating the expression of *Grin1* in the cardinal of MGE-derived interneuron subtypes. **(D)** UMAP representation colored by brain-region, highlighting the differential enrichments of cells (brown arrows) within interneuron subsets in *Grin1^*wt/wt*^* and *Grin1^*fl/fl*^* from nCX and HPC. **(E)** Stacked-barplots representing the proportions of recovered cell numbers within **(Ei)**, pooled cardinal MGE subtypes, **(Eii)**, SST subtypes; **(Eiii)**, PVALB subtypes, and **(Eiv)**, NGFC subtypes in *Grin1*-WT and *Grin1*-null from neocortex or hippocampus.*x*^2^, Chi-square test of proportions; ns, not significant.

We next performed integrated analyses of the MGE-*Grin1^*wt*^* and MGE-*Grin1^*fl/fl*^* cortical and hippocampal scRNAseq datasets. Applying similar unbiased clustering parameters used for the MGE-*Grin1^*wt*^* analyses, we observed a total of twelve *Gad1/Gad2* positive clusters in the integrated dataset (Figures 2A,B). As a robust control, *Grin1* appeared to be absent or vastly reduced in all MGE subsets in both brain regions from MGE-*Grin1^*fl/fl*^* (Figure 2C), but not in the *Slc17a7* expressing glutamatergic neurons (Figure 2-Supplement 4A). Overlaying the WT and NULL datasets from the brain regions revealed differential enrichments among the recovered cells between the genotypes (Figure 2D). Intriguingly, *Grin1*-ablation did not seem to alter the SST or PV recovery percentages, with the exception of a modest increase in the cortical NGFCs (χ^2^ = 11.6, *p* = 0.003), but not hippocampal NGFCs (χ^2^ = 4.07, *p* = 0.13) (Figure 2Ei and Figure 2-Supplements 2Ai,B). To independently examine whether *Grin1* ablation impacts interneuron abundances, we conducted immunostaining experiments to probe the PV and SST subtypes from postnatal days (PD) 30 brains from both genotypes. First, we observed no change in the total TdT^+^ cell counts from both cortex and hippocampus (Figure 2-Supplements 1Ai,Bi). Next, while we observed no change in hippocampal expressed total PV/SST cell type counts at PD30 (Figure 2-Supplement 1Bii), we observed a modest reduction in cortical PV cell type counts along with an increase in cortical SST cell type counts at the same age (Figure 2-Supplement 1Aii). This indicated differential impact of *Grin1*-ablation on cortical and hippocampal interneurons.

Despite observing no major changes in the recoveries of cardinal MGE-interneuron subtypes by scRNAseq, we observed marked changes in the recovery percentages of the subsets of SST, PV and NGFCs from both cortex and hippocampus (Figures 2Eii-iv and Figure 2-Supplements 2Aii,C). Particularly, we observed a robust increase in cortical *Chodl*-expressing cortical SST.1 population, hippocampal *Reln*-expressing SST2-4 populations, and a decrease in hippocampal SST.6 population in MGE-*Grin1^*fl/fl*^* (nCX, HPC: χ^2^ = 286, 209; *p* = 2.2e-16 for both regions). In addition, we found a reduction in the cortical PVALB.1 population, and a compensatory increase in PVALB.2/3 populations in MGE-*Grin1^*fl/fl*^* (nCX, HPC: χ^2^ = 236, 8.4; *p* = 2.2e-16, 0.14). Finally, we observed an increase in the NGFC.1 along with a compensatory decrease in NGFC.2 in both cortex and hippocampus (nCX, HPC: χ^2^ = 13, 232; *p* = 0.0003, 0.14). Among the differentially enriched subclusters, *Pthlh*-expressing PVALB.3 is quite notable (Figure 2B and Figure 2-Supplements 3A,B). This cortex-enriched cluster lacking in the hippocampus was identified within the PVALB.2 putative-chandelier cells in the MGE-*Grin1^*wt/wt*^* (Figure 1E and Figure 1-Supplement 5D), However, subsequent to integration of the MGE-*Grin1^*fl/fl*^* scRNAseq dataset, it segregated as a unique cluster, far from other PVALB clusters in the UMAP space. We observed robust expressions of genes associated with NGFCs such as *Hapln1* and *Reln* expression in PVALB.3 (Figure 2-Supplements 3A,B). Also, we observed an increase in recovery of the cortical PVALB.3 cell numbers, including the emergence of these cells in the hippocampus subsequent to *Grin1*-ablation (Figure 2-Supplements 2A,B).

### Increased SST and NGFC Subtypes in the Juvenile Brain Due to Loss of *Grin1*

**FIGURE 3:**
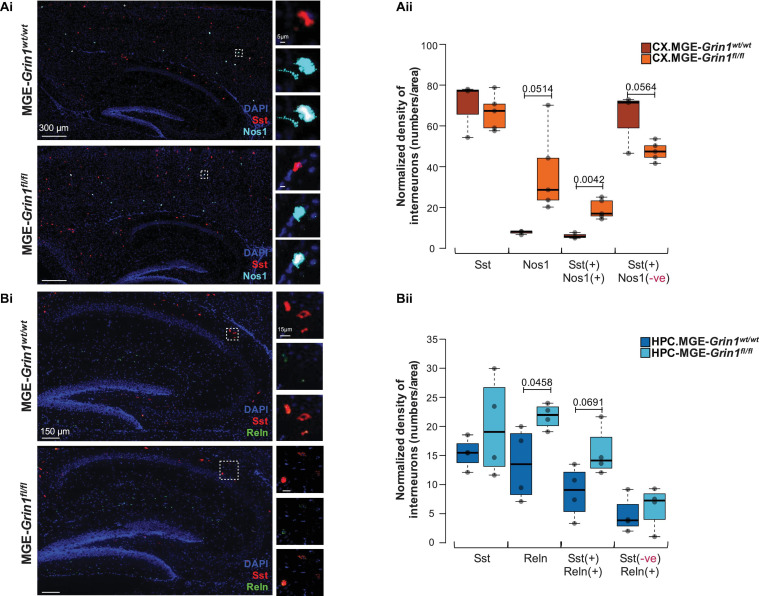
Validation of SST interneuron subtype abundances subsequent to *Grin1*-ablation by RNA *in situ* hybridization. **(A)** Examination of cortical *Nos1*-expressing *Chodl*-SST.1 subtype abundances by **(Ai)** *in situ* hybridization using *Sst* and *Nos1* RNAscope probes from P20-25 somatosensory cortex, counterstained with DAPI. **(Aii)** Boxplots indicate *Sst*(+), *Nos1*(+), *Sst*(+):*Nos1*(+) or *Sst*(+):*Nos1*(–) cell counts. **(B)** Examination of hippocampal *Reln*-expressing SST.2-4 subtype abundances by **(Bi)** *in situ* hybridization using *Sst* and *Reln* RNAscope probes from P20-25 hippocampus, counterstained with DAPI. **(Bii)** Boxplots indicate *Sst*(+), *Reln*(+), *Sst*(+):*Reln*(+) or *Sst*(–):*Reln*(+) cell counts. *n* = 4–6 brains from each genotype for immunostaining; *n* = 2 brains (4–6 sections/brain) from each genotype for RNAscope. Error bars reflect SEM; two-tailed unpaired *t*-test, for statistical analysis.

To independently establish whether the predicted differences in cell recovery percentages among the cardinal MGE subtypes are true, we conducted RNAscope *in situ* hybridization assays from PD20-25 cortex and hippocampus from both genotypes. We particularly focused on the subtypes of SST interneurons, namely the high-*Nos1*-expressing *Chodl*-SST.1 and the *Reln*-expressing SST.2-4 subtypes. First, similar to our scRNAseq prediction, we observed a significant increase in cortical *Sst*^+^ cells that co-express *Nos1* and a concomitant reduction in the *Sst*^+^ cells that lack *Nos1* after *Grin1*-ablation in MGE-derived interneurons (Figures 3Ai,ii). Next, similar to our scRNAseq prediction, we observed an increase in hippocampal *Sst*^+^ cells that co-express *Reln* (Figures 3Bi,ii), without changes in total *Sst*^+^ cell numbers in both brain regions.

**FIGURE 4:**
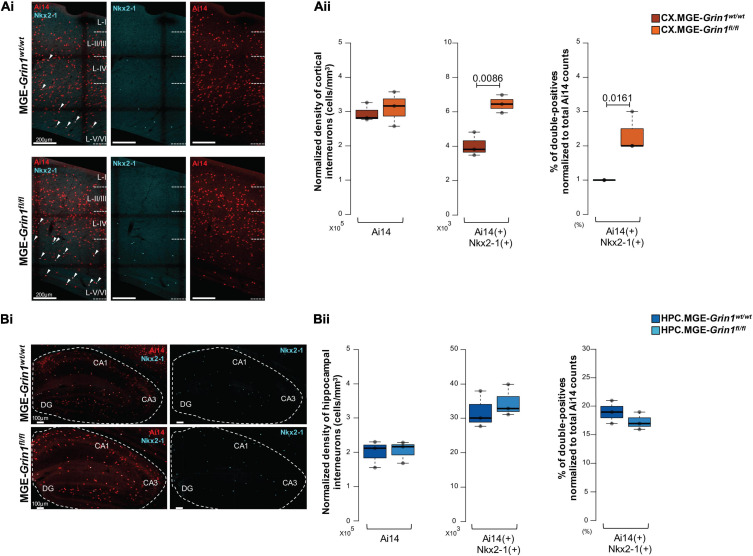
Validation of Nkx2.1-expressing interneuron abundances subsequent to *Grin1*-ablation by immunostaining. **(Ai)**, Representative immunostaining of P25-30 somatosensory cortex using anti-Nkx2-1 (cyan), and endogenous Ai14 reporter expression. **(Aii)** Boxplots indicate the normalized density of cortical Ai14(+) and Ai14(+): Nkx2-1(+) double-positive cell counts. **(Bi)** Representative immunostaining of P25-30 hippocampus using anti-Nkx2-1 (cyan), and endogenous Ai14 reporter expression (red). **(Bii)** Boxplots indicate the normalized density of hippocampal Ai14(+) and Ai14(+): Nkx2-1(+) double-positive cell counts. *n* = 3 brains (4 sections/brain) from each genotype for immunostaining; Error bars reflect SEM from individual sections; two-tailed unpaired *t*-test, for statistical analysis.

Next, we set out to examine whether the subtypes of PV and NGFC interneurons also exhibit such differences amongst their subtypes following *Grin1* ablation. We were particularly intrigued by the expression of *Nkx2.1* marker gene in subtypes of PV.3 and NGFC.1 from our scRNAseq data at ∼P20. Nkx2.1 protein is established to be expressed in cycling-MGE progenitors around ∼ED10.5 and it is thought to be turned-off in the postnatal cortex. However, it was recently indicated that subsets of late-born axo-axonic interneurons (He et al., 2016), and subsets of NGFC interneurons (Valero et al., 2021) may continue to express Nkx2.1 protein in the postnatal mouse cortex. Because both PV and NGFC interneuron subsets that express *Nkx2-1* gene appeared to be having increased cell recoveries in our scRNAseq assay, we examined the expression of Nkx2-1 protein in the postnatal brain with a verified antibody. First, anti-Nkx2-1 immunostaining revealed a clear expression of putative-nuclear staining in the cortex and the hippocampus, albeit at a lesser density than in the adjacent striatum on saggital sections. In the cortex, Nkx2-1-signal was observed frequently in the deep layers5/6 and less in the superficial layers, and hippocampal Nkx2-1-signal were observed frequently in the *stratum laconosum-moleculare* and *stratum oriens* and less in other layers (Figure 4-Supplement 1Ai,ii). While the Nkx2-1 expressing cells were all Ai14^+^, the proportion of Ai14-cells expressing Nkx2-1 was significantly higher in the hippocampus in comparison with the cortical regions. Next, co-immunostaining of anti-Nkx2-1 with anti-PV or anti-SST antibodies revealed that these Nkx2-1 cells were not double-positive with either PV or SST in both brain regions in MGE-*Grin1^*wt/wt*^* sections (Figure 4-Supplements 1B,Ci). However, we were able to observe as few as 4 cells that were double-positive for both PV and Nkx2-1 from the hippocampus of MGE-*Grin1^*fl/fl*^* across all sections counted (4 sections/animal and 3 animals/genotype) (Figure 4-Supplement Cii). While we cannot discount the possibility that some striatal *Nkx2-1^+^* cells could have polluted the scRNAseq datasets, independent immunostaining validation indicates that PV-labeled Nkx2-1 cells are indeed rare, and they could be erroneously increased upon loss of *Grin1* in the hippocampus. Since the axo-axonic cells are very weak PV or PV-negative when assayed by immunostaining (Gallo et al., 2020), one cannot discount that some proportion of these cells that indeed expresses Nkx2-1, and were missed during our counting. However, since the Nkx2-1^+^ cells in MGE-*Grin1^*wt/wt*^* sections lack PV/SST, it indicates that the majority of these cells are likely MGE-derived NGFCs, which are well established to be abundant in the hippocampus than in the cortex (Tricoire et al., 2011; Pelkey et al., 2017; Krienen et al., 2020).

Because we predicted an expansion of cortical NGFC.1 following loss of *Grin1*, we anticipated an increased proportion of Nkx2-1^+^ cells in the null tissue. Unsurprisingly, we observed a significant increase in the total numbers of Ai14^+^:Nkx2-1^+^ and also an increased in the percentage Nkx2-1^+^ cells as a proportion of total Ai14^+^ cells in the MGE-*Grin1^*fl/fl*^* cortex compared with MGE- *Grin1^*wt/wt*^* sections (Figure 4A). But, despite observing increased cell recoveries of hippocampal NGFC.1 in the scRNAseq following loss of *Grin1*, we could not observe significant changes to the hippocampal Ai14^+^:Nkx2-1^+^ double positives from the saggital sections from the *Grin1*-null tissue (Figure 4B). Since the hippocampal expressed-MGE-NGFCs are more densely expressed in the ventral hippocampus (unpublished observations, McBain lab), it is also possible that our quantitative cell counts from mid-dorsal hippocampal sections might have missed such an expansion in the hippocampus. It is unclear whether the changes in subtype numbers reflect a change in cell identity or whether this is altered subtype proportions due to differential survival. Nevertheless, our independent examination strongly supports the role for *Grin1*-mediated alterations in MGE-derived interneuron subtype abundances in the juvenile cortex.

### NMDAR Signaling Shapes the Transcriptional Landscape in MGE-Derived Interneurons

**FIGURE 5:**
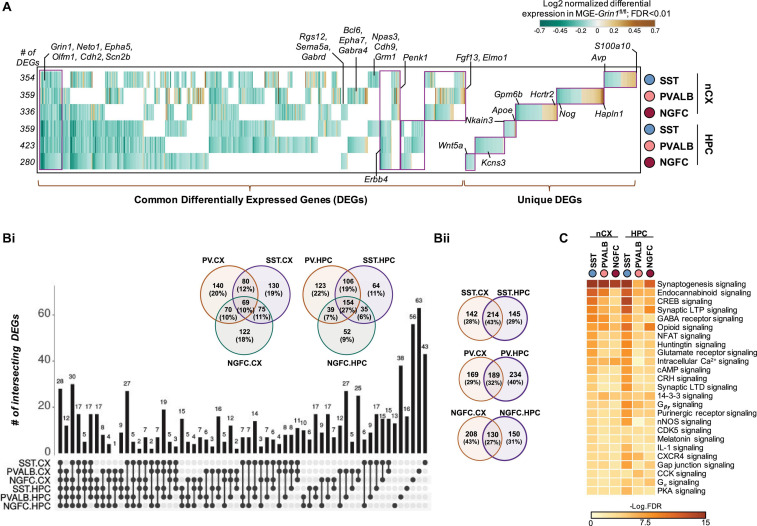
Cell-autonomous transcriptional changes subsequent to MGE-specific developmental *Grin1*-ablation. **(A)** Combined heatmap representing the 802 differentially expressed (DEGs) in the neocortical and hippocampal MGE-derived interneurons upon *Grin1*-ablation, at a FDR < 0.01 and FC > 10%, as determined by MAST analysis (see details in section “Materials and Methods”). **(Bi)** UpSet plot and Venn-diagrams, respectively indicating the intersection and percentages of DEGs common across MGE subtypes from neocortex or hippocampus. **(Bii)** Venn-diagrams indicating the percentages of DEGs common within individual MGE subtypes from neocortex and hippocampus. **(C)** Ingenuity Pathway Analysis of significantly overrepresented molecular pathways in each MGE-subtype. FDR determined by Fisher’s Exact Test.

It is now well-established that transcriptional signatures define the subtype identities of GABAergic interneurons (Fishell and Kepecs, 2020). To examine the full range of transcriptional impairments triggered by *Grin1* ablation in MGE-derived interneurons, we next performed differential gene expression testing by pooling the SST/PVALB/NGFC subtypes into their cardinal MGE classes to identify the genes that are differentially expressed between the genotypes. For instance, SST1-6 and TH.1 are pooled together as SST; PVALB1-3 are pooled together as PVALB, and NGFC1-2 are pooled together as NGFC cardinal classes for this assay. At a stringent false-discovery rate (FDR) < 0.01, 802 genes passed the 10%-fold-change (FC) threshold across the MGE subtypes from both brain regions (Figure 5A and Supplementary Table 1). Several interesting features were observed in the differentially expressed gene (DEG) pattern upon MGE-specific *Grin1*-ablation. *(i)* Among all DEGs only ∼10 and 1% are upregulated in the cortex and hippocampus, respectively, while the remaining genes were all downregulated (Figure 5-Supplements 1Aii,B,C). *(ii)* While *Grin1* ablation resulted in several unique DEGs between the MGE classes, ∼10 and 27% of the DEGs are common within cortex and hippocampus, respectively (Figures 5A,B). For instance, while *S100a10*, *Hapln1*, *Hcrt2* are uniquely upregulated in cortical SST, PV, and NGFC, respectively (Figure 5A), *Apoe*, *Kcns3*, *Wnt5a* were uniquely altered in hippocampal SST, PV, and NGFC, respectively. In contrast, *Grin1* ablation induced common changes in *Penk1* and *Erbb4* expression patterns across all MGE-derived interneuron classes in the cortex and hippocampus, respectively. *(iii)* ∼27–43% of all DEGs were shared by MGE classes across brain regions (Figure 5B). For example, *Npas3*, *Cdh9*, *Grm1* are commonly downregulated in all SST subclasses; *Bcl6*, *Epha7*, *Gabra4* common to PV class; and *Rgs12*, *Gabrad, Sema5a* common to NGFCs from both brain regions. *(iv)* Lastly, 28 genes are commonly differentially expressed across both brain regions, across all MGE subtypes. For example, *Grin1*, *Neto1*, *Cdh2*, *Scn2b* are commonly downregulated across the board, while *Epha5*, *Olfm1* are commonly downregulated across all, but cortical PV cells (Figure 5A).

To examine the broad biological impact of the DEGs, we performed Gene Ontology (GO) analyses. Broad GO analyses on all DEGs indicates that these genes serve to regulate multiple molecular functions in interneurons, including regulation of GABAergic and glutamatergic synapses, additional to biological pathways related to addiction and circadian entrainment (Figure 5-Supplement 2A). Further classification of DEGs based on their cellular functions within the MGE subtypes revealed genes critical for regulation of membrane excitability, gene expression, synaptic partnering and assembly, as well as major intracellular Ca^2+^ signaling cascades, second messengers and regulators of cellular energetics (Figure 5C, Figure 5-Supplement 2B, and Supplementary Table 2).

### Transcription Factor Expression Is a Key Component of NMDAR-Mediated Regulation of MGE-Derived Interneurons

**FIGURE 6:**
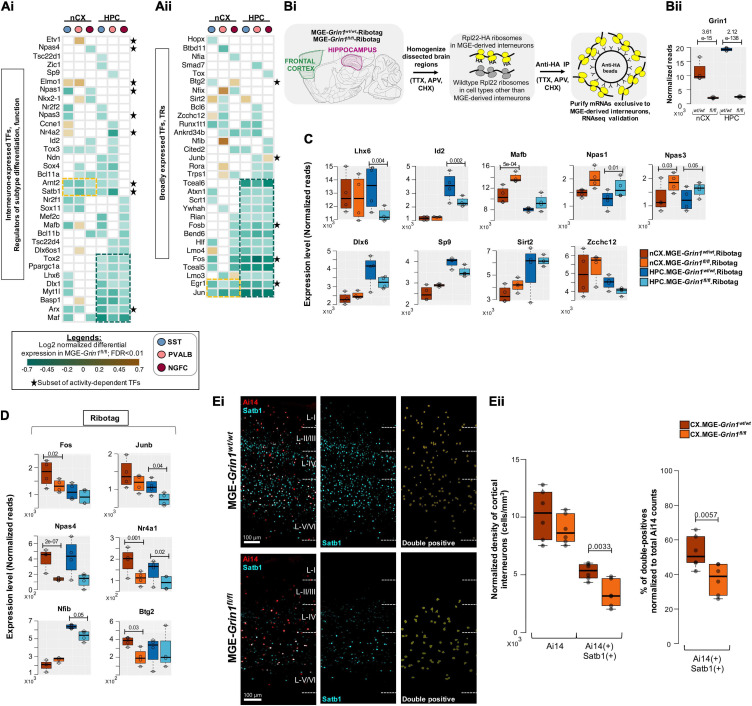
NMDAR signaling in MGE-derived interneurons are highly dedicated to the transcriptional control of interneuron identity. **(A)** Heatmap of log2 FC of significant DEGs in neocortical and hippocampal MGE cardinal subtypes, showing a subset of **(Ai)**, Transcription factors (TFs) that are previously established to regulate MGE subtype identity and function; **(Aii)**, broadly expressed TFs and transcriptional regulators (TRs) that are not currently known to regulate MGE function. Clusters of commonly differentially expressed genes in neocortex and hippocampus are indicated in yellow or green boxes. * indicates a subset of neuronal activity-regulated TFs. **(Bi)** Overview of the experimental workflow for RNAseq validation, by ribosomal tagging/Ribotag strategy specific to MGE-derived interneurons. *n* = 4 biological replicates from each genotype for MGE-Ribotag-seq; Error bars reflect SEM; DeSeq2 for statistical analysis. **(Bii)** MGE-Ribotag-seq normalized reads for *Grin1* expression in neocortex and hippocampus. MGE-Ribotag-seq normalized reads cross-validating the differential expressions of **(C)**, MGE-enriched TFs, and **(D)**, neuronal activity-regulated TFs. **(Ei)** Representative immunostaining of P25-30 somatosensory cortex using anti-Satb1 (cyan), and endogenous Ai14 reporter expression. Ai14:Satb1 double-positive cells are represented as yellow surfaces using Imaris9.6. **(Eii)** Boxplots indicate the normalized density of cortical Ai14(+) and Ai14(+): Satb1(+) double-positive cell counts. *n* = 3 brains from each genotype for immunostaining; Error bars reflect SEM from individual sections; two-tailed unpaired *t*-test, for statistical analysis.

Because transcriptional regulation underlies numerous fundamental processes including the expression of other classes of genes, we next examined the DE-transcriptional regulators in detail. We first examined the 67 genes that are differentially expressed upon *Grin1*-ablation and are known to mediate transcriptional regulation of gene expression. Of these, 35 genes are previously established to be expressed in different GABAergic interneuron classes including some notable MGE-expressed transcription factors (TFs) (Figure 6Ai). The remaining 32 are broadly expressed TFs (Figure 6Aii), that include a small subset of 15 genes that are regulated by neuronal activity. Barring a few genes, we observed the majority of TFs to be down regulated in both brain regions. Intracellular Ca^2+^ signaling cascades and second messenger systems are key mediators of NMDAR signaling to the nucleus for transcriptional regulation. Theoretically, an early first wave impairment of Ca^2+^ signaling in *Grin1*-lacking MGE progenitors could result in transcriptional silencing of the mediators of Ca^2+^ signaling cascades and second messenger systems, which would sustain the transcriptional impairments. Indeed, we also observed a downregulation of various Ca^2+^ homeostasis-regulators, kinases, phosphatases and second messengers that are activated downstream of NMDAR signaling, including regulators of cellular energetics and mitochondrial function (Figure 6-Supplements 1A–D). Furthermore, we noted that hippocampal MGE neurons had a greater proportion of DE-TFs and kinase signaling cascade effectors that were downregulated across all 3 subtypes compared to their cortical counterparts. Together, this suggests that hippocampal MGE-derived interneurons may be more vulnerable than cortical MGE-derived interneurons toward *Grin1*-mediated Ca^2+^ transcriptional silencing at this age.

Interestingly, among the early TF cascades in the progenitors that sequentially determine and maintain MGE fate, several members appear to be expressed at ∼P20, and starkly downregulated upon *Grin1*-ablation. For instance, *Lhx6*, *Maf*, *Arx*, *Myt1l*, *Dlx1* are among the genes broadly downregulated across all hippocampal MGE subtypes and within specific class(es) in their cortical parallels (Figure 6A). Other MGE fate-determining TFs, *Nkx2-1*, *Mafb*, *Satb1*, *Nr2f1* (*CoupTf1*), *Sp9*, also appear to be downregulated in discrete populations. This also includes a downregulation of *Bcl11b* (*Ctip2*) in both hippocampal and cortical NGFCs, a gene recently linked to regulation of NGFC morphology and function (Nikouei et al., 2016). Among the few transcriptional regulators upregulated are *Sirt2*, *Elmo1*, *Zcchc12*, none of which have been extensively characterized by overexpression or knockout studies to examine their specific roles within MGE-derived interneurons (Figure 6B). *Sirt2* is an established transcriptional repressor (Erburu et al., 2017) that may regulate the repression of several target genes in an MGE-specific manner. *Elmo1* has been previously characterized during the activity-dependent migration of CGE subtypes (De Marco García et al., 2011), and more recently predicted to be a candidate marker of immature *Pvalb* + interneurons in the hippocampus (Ling-Lin Pai et al., 2020). Finally, a recent study has predicted that the expression of *Zcchc12* correlates with slower intrinsic firing among hippocampal CA1 interneurons (Harris et al., 2018). This suggests that increased *Zcchc12* expression might regulate the expression of synaptic genes enabling reduced intrinsic excitability in the MGE subsets. Related to such putative decreased excitability in the MGE-derived interneurons, among the activity-regulated TFs, we observe broad downregulation of *Jun*, *Egr1*, *Fos*, *Fosb*, *Arc*, *Satb1*, *Arnt2* across all classes of MGE in both brain regions (Figures 6A,B, indicated by ^∗^), and a subtype-specific down regulation in *Npas4*.

### MGE-Ribotag-Seq Cross-Validates Impaired Transcription Factor Expressions Due to MGE-*Grin1*-Ablation

To independently validate the changes in gene expressions observed by scRNAseq, we employed a Ribotag-based strategy where we generated a triple-transgenic mice by breeding the *Nkx2.1^*cre*^*:*Grin1^*fl/fl*^* with the *Rpl22^*HA/HA*^*-containing Ribotag mice (Sanz et al., 2009), hereafter called MGE-Ribotag (Figure 6Bi and Figure 6-Supplement 1E). We recently established that this is a robust tool to examine the mRNAs associated with the translational machinery, in a manner exclusive to MGE-derived GABAergic interneurons (Mahadevan et al., 2020). mRNAs copurified with anti-HA immunoprecipitation were utilized to prepare cDNA libraries that were subsequently sequenced in order to investigate changes in gene expression changes resulting from MGE-specific *Grin1*-ablation. By utilizing cortical and hippocampal tissues from age-matched MGE-Ribotag from both genotypes, we first established a robust reduction in *Grin1* expression in both brain regions (Figure 6Bii). Similar to the observed changes in the scRNAseq assay we observed robust changes in several key TFs by MGE-Ribotag-seq, particularly in genes that have established functions in maintaining MGE-identities. Notably, we observed a significant downregulation of hippocampus expressed *Lhx6* and *Id2*, an upregulation in cortical *Mafb* and hippocampal *Npas1*, and an increased expression of *Npas3* in both brain regions, after *Grin1* ablation (Figure 6C, top).

Key differences exist between the two strategies scRNAseq and MGE-Ribotag-seq. The scRNAseq assays only the mRNAs present in the neuronal soma while providing a single-cell resolution across the subtypes of MGE-derived interneurons. The MGE-Ribotag-seq, however, lacks the single-cell resolution across MGE-interneuron subtypes but includes the mRNAs that are bound to the translational machinery and much closer to the cellular proteome. Moreover, MGE-Ribotag-seq includes the translating-mRNAs present in the neuronal processes. Despite these technical differences, the congruity in the DEGs after *Grin1*-ablation strongly favors our claims related to NMDAR-signaling dependent regulation of key transcription factors. Having a gene not validated by MGE-Ribotag-seq does not imply a false-positive scRNAseq result, and the above-described technical differences could contribute to it. Also, the genes that are commonly differentially expressed across the PV-SST-NGFC subtypes by scRNAseq will have a greater possibility of being identified as a DEG via MGE-Ribotag-seq. Accordingly, genes such as *Dlx6*, *Sp9*, *Sirt2*, and *Zcchc12* that showed alternating differences among subtypes in the scRNAseq, did not reveal significant differences via MGE-Ribotag-seq (Figure 6C, bottom).

We also observed a significant decrease in the expressions of several activity-regulated TFs by MGE-Ribotag-seq in both brain regions (Figure 6D). While *Fos*, *Junb*, *Btg2*, *Nfib* expressions were significantly downregulated in either cortex or hippocampal MGE-derived interneurons, the expressions of *Nr4a1* and *Npas4* appear to be commonly downregulated in both brain regions after *Grin1*-ablation. *Satb1* is a key activity-regulated TF that is expressed in MGE-derived interneurons, critically regulating the identity and survival of different subtypes (Close et al., 2012; Pachnis et al., 2012). Independent immunostaining experiments revealed that *Grin1*-ablation resulted in a robust reduction in MGE-derived interneurons co-expressing Satb1 in PD30 somatosensory cortex (Figures 6Ei,ii), further substantiating our findings from scRNAseq. Taken together, this establishes a framework for simultaneous regulation of neuronal activity and expression of distinct sets of TFs (including the activity-regulated TFs) by NMDAR-signaling in MGE-derived interneurons.

### Impaired NMDAR Signaling Alters Region-Specific MGE Subtype Marker Expression

**FIGURE 7:**
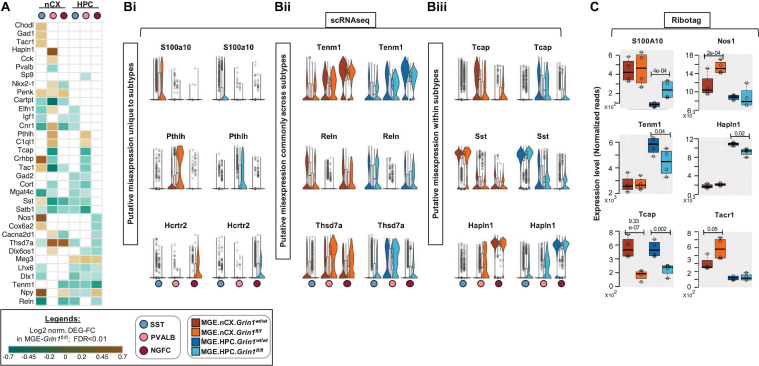
Differential expression of interneuron marker genes across subtypes upon *Grin1*-ablation. **(A)** Heatmap of log2 FC of significant DEGs in neocortical and hippocampal MGE cardinal subtypes, showing notable MGE marker genes. Representative box-violin plots of top differentially expressed genes from the above that represent **(Bi)**, a misexpression unique to a single MGE-interneuron subtype in the *Grin1^*fl/fl*^* **(Bii)**, a misexpression across all MGE-derived interneuron subtypes in the *Grin1^*fl/fl*^*; **(Biii)**, a misexpression between MGE-derived interneuron subtypes in the *Grin1^*fl/fl*^*. **(C)** MGE-Ribotag-seq normalized reads, cross-validating the misexpressed interneuron marker genes in neocortex and hippocampus.

Several GABAergic MGE markers were misregulated upon *Grin1*-ablation (Figure 7A). For example, genes *S100a0, Pthlh, Hcrtr2* that are normally expressed in SST, PV and NGFCs, respectively, are upregulated in the same clusters of MGE-*Grin1^*fl/fl*^* (Figure 7Bi), indicating a misexpression in a subtype-specific manner. Next, while certain genes such as *Reln*, *Tenm1* are broadly downregulated across MGE classes, some genes like *Thsd7a* show an upregulation in certain classes but a downregulation in the other classes (Figure 7Bii). Interestingly, a few genes that are normally abundant in one MGE class, appear to be misexpressed in another MGE class where they are not abundant. For instance, *Tcap (telethonin)*, that is expressed in PV cells, in addition to being decreased in PV cells, is upregulated in NGFCs in both cortex and hippocampus. Similarly, *Hapln1* expression, which appears to be abundant in NGFCs (Harris et al., 2018; Krienen et al., 2020), is upregulated in PV subsets (Figure 7Biii). Indeed, MGE-Ribotag-seq cross-validates a robust downregulation in *Tcap*, *Tenm1*, and *Hapln1*, and a robust upregulation in *S100a0*, *Tacr1*, and *Nos1* (Figure 7C). Lastly, we observed an upregulation in the *Gad1* and *Slc32a1* (vesicular GABA transporter, vGAT) and a downregulation in *Gad2* and *Slc6a1* (Na^+^-Cl^–^ dependent GABA transporter, GAT1), corresponding with GABA synthesis and reuptake machineries, respectively (Figure 7-Supplement 1A). These findings closely match the trending differences observed via MGE-Ribotag-seq.

### NMDAR Signaling Regulates MGE Subtype-Specific Expression of Neurodevelopmental Disorder Risk Genes

**FIGURE 8:**
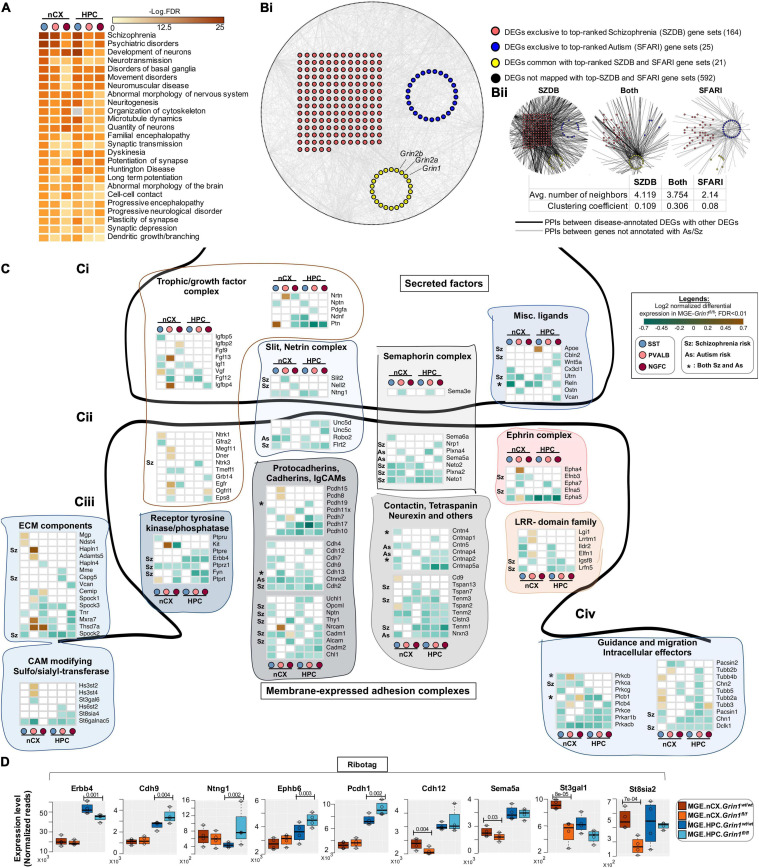
Aberrant NMDAR signaling results in misexpression of high-risk Sz genes. **(A)** Ingenuity Pathway Analysis of significantly overrepresented disease pathways in each MGE-subtype. FDR determined by Fisher’s Exact Test. **(Bi)** Global protein-protein interaction (PPI) map among all differentially expressed genes (DEGs). Red circles indicate the DEGs annotated to be top-ranked Sz-risk genes; Blue circles indicate the DEGs annotated to be top-ranked As-risk genes; Yellow circles indicate the DEGs annotated with both Sz and As-risk genes. Black circles in the periphery indicate the DEGs not annotated with high-risk Sz/As genes. The PPIs between DEGs indicated in gray lines. **(Bii)** PPIs between Sz/As/dually enriched clusters, and other genes. The PPIs between disease-annotated DEGs and other disease-annotated DEGs or with other non-annotated DEGs are indicated in black lines. The PPI between non-annotated DEGs indicated in gray lines. **(C)** Heatmap of log2 FC of significant DEGs in cortical and hippocampal MGE cardinal subtypes, showing a subset of **(Ci)**, secreted trophic factors and secreted ligands and guidance cues. **(Cii)** Membrane-bound synaptogenic receptors and cell adhesion molecules (CAMs) **(Ciii)**, extracellular Matrix (ECM) components and matrix modifying enzymes. **(Civ)** Intracellular effectors of guidance and synaptogenic cues. **(D)** MGE-Ribotag-seq normalized reads, cross-validating the differentially expressed synaptic partnering molecules.

Interneuron-centric disease etiology is an emerging centrality in multiple psychiatric disorders (Marín, 2012). Thus, we questioned whether the *Grin1* ablation induced DEGs presently identified correlate with disease etiology. Disease-ontology based Fisher’s Exact Test conducted on the DEGs showed significant over-representation of genes implicated in “Schizophrenia,” “Psychiatric disorders” and “Movement disorders,” among other cellular impairments involving aberrant morphology of neurons (Figure 8A and Supplementary Table 3). To independently examine the DEGs for potential enrichment for neurodevelopmental disorders, we obtained the risk genes for schizophrenia (Sz) and autism spectrum (As) from the SZDB (Wu Y. et al., 2017) and SFARI (Simons Foundation, 2018) databases, respectively. These databases curate and rank disease-relevant gene sets, based on multiple evidence sources including genome-wide association studies, copy-number variations, known human mutations and other integrative analyses. In particular, we mapped the DEGs with the top-ranked genes from these disease datasets (see methods for details). While 592 DEGs could not be mapped with either disease genes, 25 genes mapped exclusively with the SFARI-AS gene list, 164 genes mapped exclusively with the SZDB-Sz gene list and 21 genes mapped with both datasets (Figure 8Bi and Supplementary Table 4). It is now well-established that several neurodevelopmental disorders exhibit a high degree of converging molecular pathways employing proteins that exist in physical complexes (Cristino et al., 2014). Therefore, we examined whether these disease-associated DEGs are known to form protein complexes between each other, by mapping curated protein-protein interaction (PPI) datasets for all 802 DEG products. Indeed, we observed that > 95% of disease annotated DEG products are known to exist with PPIs, while only ∼75% of DEG products not annotated with Sz/As are known to exist with PPIs (Supplementary Table 4C). Interestingly, despite not mapping directly with the high-ranked disease gene sets, the remaining 592 genes are observed to exist in tightly knit PPIs with the disease annotated genes. However, the PPIs mapped with SZDB form the most interconnected clusters in comparison to the SFARI-mapped PPI network (Figure 8Bii), as indicated by relatively higher clustering coefficient. Put together, both unbiased disease ontology prediction, targeted analysis of high-confidence neuropsychiatric disorder genes, indicate that members of the DEGs share physical, and functional pathways in MGE-derived interneurons, contributing toward disease etiology.

Among the 210 DEGs mapped with to Sz and As, 45 genes are established regulators of axon pathfinding, synapse formation and assembly, while 38 members are established regulators of membrane excitability and neuronal firing. Because both gene classes are intimately associated with interneuron function, we examined these classes in detail. We observed multiple classes of secreted ligands and cognate receptor families corresponding to semaphorin, netrin, slit, chemokine and growth factors, and their intracellular effectors that are downregulated upon MGE-*Grin1*-ablation (Figures 8Ci,iv). These include *Ntng1*, *Sema3e*, *Slit2*, *Cx3cl1*, and some of their receptors, *Unc5c*, *Nrp1*, *Neto1/2*, *Robo2* that are decreased in MGE-class-specific manner. We observed *Fgf13* that was recently demonstrated to mediate MGE-subtype specific synapse assembly, to be upregulated in cortical PV cells, but downregulated in cortical SST, while *Apoe* to be upregulated in hippocampal SST cells. In addition to synaptic assembly molecules, we observed DE in a variety of synaptic adhesion molecules, corresponding to protocadherin, cadherin, ephrin, and contactin families (Figure 8Cii). Notably, we also observed a downregulation of *Erbb4* across all hippocampal MGE-subtypes. Lastly, we observed increased expression of extracellular matrix components *Mgp*, *Ndst4*, *Hapln1*, *Adamts5*, *Mxra7, Thsd7a*, and the matrix modifying enzymes *Hs3st2/4* in cortical SST/PV subtypes (Figure 8Ciii). In parallel, MGE-Ribotag-seq cross-validates a robust down regulation in hippocampal *Erbb4*, and a robust differential expression of several synaptic partnering and adhesion molecules including *Cdh9*, *Ntng1*, *Ephb6*, *Pcdh1*, *Cdh12*, *Sema5a*, and key members of CAM modifiers *St3gal1* and *St8sia2* (Figure 8D).

Among the regulators of neuronal excitability, we observed a downregulation of multiple members of postsynaptic glutamate receptor subunits, GABA receptors and their associated partners (Figure 8-Supplement 1Bii). Interestingly, while we noted a broad downregulation of several members of potassium and sodium channel subunits, a few discrete members of the Kcn-families were upregulated in cortical PV and NGFC subtypes. Finally, we also observed multiple members of presynaptic GABA synthesis, release and uptake machineries including *Gad1, Syt2/10*, and *Slc6a1* differentially expressed in discrete MGE subtypes (Figure 8 and Figure 8-Supplement 1Bi). Collectively, these findings highlight the centrality of MGE-expressed *Grin1*-signaling during synapse formation and connectivity, which when aberrantly expressed, can lead to neurodevelopmental disorders.

## Discussion

### Centrality of MGE-Derived Interneuron-Expressed NMDARs From Juvenile Brain

**FIGURE 9:**
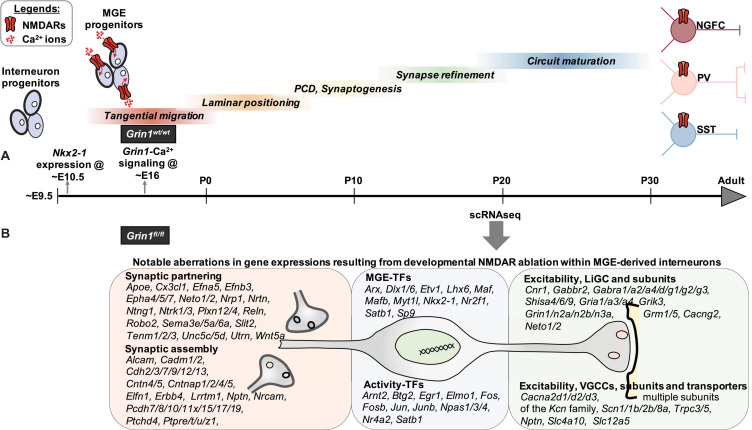
Transcriptional control of MGE-derived interneuron development, synaptic partnering, and excitability are mediated by NMDAR signaling. **(A)** *Nkx2.1* expression appears at ∼ED10.5, driving subtype fate in MGE-expressed interneuronal progenitors. Subsequently, their sequential developmental milestones toward circuit refinement appears to be under a combination of innate genetic mechanisms and neuronal activity. While the earliest *Grin1* expression is reported at ∼ED14 in developing brain, MGE-specific NMDAR mediated Ca^2+^ is recorded at ∼ED16. However, the broad role played by interneuron-expressed NMDAR signaling during interneuron development and maturation until now is not well delineated. **(B)** By driving *Grin1*-ablation using *Nkx2.1*-driven Cre-recombinase expression, we report the earliest developmental loss of NMDAR signaling, across MGE-derived interneuron subtypes. In particular, by performing scRNAseq assay in MGE-derived interneurons from the neocortex and hippocampus of juvenile mouse brain, we report a broad transcriptional aberration subsequent to loss of NMDAR-signaling. Notably, this expression abnormality involves numerous TFs, synaptogenic and regulators of interneuron excitability, that collectively establish MGE subtype identities and drive their maturation. (TF, transcription factors; LiGC, Ligand-gated ion channel; VGCC, Voltage-gated ion channel).

NMDARs serve as critical activity dependent signaling hubs for myriad neuronal functions due to their innate ability to directly link network dynamics to cellular calcium events and associated transcriptional coupling. Such NMDAR-dependent E-T coupling is widely established in glutamatergic neurons and in specific interneurons (Wamsley and Fishell, 2017; Yap and Greenberg, 2018) using candidate approaches within mature circuits. However, the detailed unbiased evaluation of the transcriptional landscape of NMDAR signaling within interneurons in developing circuits undergoing refinement is lacking. Our study provides the first systematic “fingerprinting” of the transcriptional coupling associated with NMDAR signaling, exclusive to MGE-derived interneurons, providing a road map for examining NMDAR regulation of MGE-derived interneurons in a subtype specific manner. Our unbiased transcriptional profiling approach indicates that developmental NMDAR signaling participates in driving MGE-derived interneuron development and maturation by regulating the expression of transcription factors (67 genes), synaptogenic (53 genes) and connectivity factors/adhesion molecules (61 genes), and regulators of membrane excitability (78 genes), among the 802 DEGs in interneurons (Figure 9). While the present study reflects a longer-term impact of the developmental loss of NMDAR-signaling in MGE-derived interneurons, based on broad transcriptional downregulation of target genes, we can make several predictions that should guide future investigations.

### Role for NMDAR-Signaling Regulated TFs in Shaping Interneuron Identity and Granularity Amongst Subtypes

Three key possibilities that enable NMDAR-dependent interneuron development exists, and our dataset provides potential examples for all three scenarios. *(i)* First, it is possible that NMDAR-dependent Ca^2+^ signaling in the developing interneuron progenitors provides a combinatorial cue that will couple with innate genetic programs to generate the diversity in interneuron subtypes. We observe key MGE-specification genes *Lhx6* and *Id2* downregulated and other genes such as *Mafb*, *Npas1*, and *Npas3* upregulated in *Grin1*-lacking interneurons in both scRNAseq and MGE-Ribotag-seq assays. *(ii)* It is also possible that NMDAR signaling critically regulates neuronal activity that further shapes the expressions of MGE-subtype determining and distinct activity-dependent TFs. In line with this possibility, we establish a robust downregulation in the activity-regulated TF, *Satb1*, and a robust reduction in Satb1 + cortical interneurons that lack *Grin1*. It is notable that *Satb1* is a key activity-regulated TF that critically regulates the identity and survival of different subtypes of SST-interneurons (Close et al., 2012; Pachnis et al., 2012). Additionally, we observe an increase in *Etv1* expression in cortical PV cells, and *Etv1* was previously demonstrated to be an activity-dependent TF that inversely correlates Ca^2+^ influx (Dehorter et al., 2015), regulating the properties of a subset of PV-interneurons. *(iii)* Lastly, it is possible that altered neuronal activity due to aberrant NMDAR -signaling result in differential survival and/or cell-death in developing interneurons (De Marco García et al., 2011, 2015; Wamsley and Fishell, 2017; Denaxa et al., 2018; Priya et al., 2018). Indicating that the *Grin1*-lacking MGE-derived interneurons have altered interneuron survival and/or cell-death, we observe differential recoveries of the subtypes within SST and NGFC in the scRNAseq assay. Particularly, we observe an abundance of cortical *Chodl*-SST.1 in the *Grin1*-ablated MGE-derived interneurons, and a concomitant decrease in cortical SST.2-4 subtypes. We also observe an increase in Nkx2-1 expressing putative neocortical NGFCs in the *Grin1*-ablated MGE-derived interneurons. Moreover, we report an increase in hippocampal *Reln*-SST2-4 subtypes upon *Grin1*-ablation by RNA *in situ* hybridization. Additionally, by independent immunostaining experiments, we observe a robust decrease in cortical MGE-derived interneurons that coexpress the TF *Satb1*, which is established to promote interneuron survival. However, detailed future studies are necessary to uncover which of the above indicated NMDAR-dependent mechanisms regulate the abundances and diversity within PV/SST/NGFC subclasses.

### Shaping Interneuron Subtype-Specific Synaptic Assembly and Connectivity

What is the biological context of differential expression of the TFs, in the juvenile forebrain when MGE-derived interneuron fate is assumed to be already sealed, and subtype identities established? It is emerging that some of these TFs are continually required for the maintenance of MGE fate, post development (Fishell and Kepecs, 2020). One of the ways the TFs maintain MGE subtype fate into adulthood, is by controlling the expression of genes that are essential for ongoing interneuron function. Accordingly, we predict that NMDAR-dependent expression of synaptogenic and synaptic partnering molecules regulate the assembly of synapses with appropriate targets. Secreted semaphorin, ephrin, slit, netrin and neurotrophin-based signaling systems have been investigated in GABAergic neurons, during axonal pathfinding, and cell migration (Marín et al., 2001; Polleux et al., 2002; Andrews et al., 2008; Nóbrega-Pereira et al., 2008; Zimmer et al., 2008). However, only recently have inroads been made into delineating their expression, and interaction with appropriate receptor systems in target synapses during accurate synaptogenesis. In addition, the NMDAR-dependent expression of synaptic adhesion molecules will further promote stability of newly formed synapses. Here, the mis-expression of diverse secreted cues, their receptors and adhesion molecules by MGE subtypes during *Grin1*-ablation, provides unique insight into the molecular diversity employed during synapse establishment. Our findings also reveal numerous candidates for examining subtype specific synapse assembly, which are centrally regulated by NMDAR signaling.

After synapse formation, nascent connections remain susceptible to strength modifications according to neuronal activity. Again, NMDAR-signaling in MGE-derived interneurons seems to regulate this process by the transcriptional regulation of the expressions of both presynaptic and postsynaptic members, including excitatory and inhibitory synaptic molecules and their auxiliary subunits, as well as presynaptic GABA release machinery molecules such as *Cplx1/2, Stx1b, Rab3c*. Cell-specific ablation of *Grin1* is previously reported to regulate neuronal intrinsic excitability (Hou and Zhang, 2017). Here, we observe a broad down regulation of several members of the potassium channel subunits and their auxiliary subunits across MGE subtypes, except for an upregulation of a few *Kcn*-genes in cortical PVs and NGFCs. While the precise impact of the diverse changes in these genes on MGE firing are currently unclear, the pattern of expression of the activity-dependent transcription factors provides us an indication.

Notable activity-dependent TFs such as *Jun, Egr1* are downregulated across all MGE sub-types, while *Fosb, Fos, Arx* are down regulated across all hippocampal MGEs, and *Satb1*, *Arnt2* are downregulated across all cortical MGEs. In addition, *Npas4*, an established early response TF activated upon neuronal activity and Ca^2+^ influx in MGE-derived interneurons (Spiegel et al., 2014), was downregulated in cortical NGFCs upon *Grin1*-ablation. Lastly, *Ostn* was recently established as an activity-regulated secreted factor (Ataman et al., 2016), and we observed *Ostn* to be downregulated specifically in cortical PV subtypes. Together, these changes are consistent with reduced neuronal activity in MGE subtypes upon *Grin1*-ablation, recapitulating previous reports indicating that NMDAR-antagonists can directly reduce the activity of GABAergic interneurons in adult mice (Homayoun and Moghaddam, 2007). The differential expressions of these activity-TFs also serve as ripe candidates to examine activity-dependent survival of specific interneuron subtypes in the juvenile brain. Interpreting the differential expressions of activity-dependent genes during scRNAseq has been challenging, particularly, when these genes could get activated by the very process involved in cell dissociation and sorting (Wu Y. E. et al., 2017). However, our use of activity-blockers and actinomycin-D throughout our MGE-*Grin1^*wt*^* and MGE-*Grin1^*fl/fl*^* scRNAseq pipelines and independent validations via MGE-Ribotag-seq gives confidence that the differential expressions of activity-dependent TFs reflect biological relevance.

### NMDAR Signaling in NGFCs

Among the MGE subtypes, the PV and SST interneurons are traditionally widely studied in comparison to the dendrite-targeting NGFC subtypes (that include the Ivy cells). In the present study we provide the first detailed molecular insight into the cortical and hippocampal NGFCs, subsequent to NMDAR ablation. We anticipated that these cell types could be particularly susceptible to loss of NMDARs, since we previously reported that NGFCs exhibit the most robust synaptic NMDAR conductances among the MGE subtypes (Matta et al., 2013). While our study was being corresponded, a recent study demonstrated the existence of deep layer neocortical NGFCs that continue to express Nkx2-1 in the postnatal mouse brain (Valero et al., 2021). In the present study we establish the existence of Nkx2-1-expressing MGE-derived interneurons in the juvenile mouse cortex and hippocampus through immunostaining. Moreover, we report that neocortical but not hippocampal Nkx2-1-expressing putative NGFC abundance is regulated by NMDAR-signaling. While we conducted the cell counting across the entire saggital section, we are confident of the robustness of our neocortical Nkx2-1 cell counts. However, since we missed the ventral hippocampus in our saggital brain sections, our quantitative cell counts from mid-dorsal hippocampal sections might have missed such an expansion in the hippocampus. We also identified many TFs (*Bcl11b, Id2*) and activity-TFs (*Npas4*) differentially expressed in these NGFCs after the loss of *Grin1*, and thus our dataset provides novel targets for examining activity-dependent survival of NGFCs. Finally, NGFCs exhibited dendritic arborization impairments subsequent to impaired NMDA signaling (De Marco García et al., 2015; Chittajallu et al., 2017). Indeed, we observe 49 genes among the DEGs (Supplementary Table 1) that have established roles in regulating neuronal cytoskeleton and associated signaling. Therefore, our dataset is a robust repository for detailed examination of NGFC function by providing several candidates.

### Developmental NMDAR Ablation in Interneurons and Schizophrenia

Impaired NMDAR function observed during human NMDAR gene mutations, and anti-NMDAR-encephalitis results in a wide range of neuropsychiatric disorders including autism spectrum disorders, intellectual disability, psychosis, epilepsy and associated comorbidities (Lakhan et al., 2013). While broadly aberrant NMDAR signaling in neurons is thought to underlie a wide range of these neurological disorders, an interneuron-centric developmental NMDAR aberration is emerging central to schizophrenia-related syndromes (Nakazawa and Sapkota, 2020). Indeed, in the present study, disease mapping of the DEGs using high-ranked SZDB-Sz and SFARI-As datasets indicate that many more DEGs map with the Sz than the As database. Moreover, proteins encoded by these disease relevant DEGs exist in physical and functional complexes with the ones encoded by DEGs that are not directly mapped to the Sz database. We used only stringent, high-ranked disease genes from the database that pass several disease-relevant criteria. However, there are other DEGs that still map to lower-ranked Sz and As datasets that are “non-annotated” in present study. While our study can be argued as an “extreme” case of NMDAR hypofunction in MGE-derived interneurons, it provides a starting point highlighting the centrality and broad range of interneuronal NMDAR-transcriptional pathways during development. Several studies implicate NMDAR-hypofunction specific to PV cell types as a central underlying feature of schizophrenia etiology (Lewis et al., 2005). However, the measurable NMDAR conductances within PV interneurons are relatively small in comparison to other MGE subtypes (Matta et al., 2013). In addition, *Pvalb*^*Cre*^-driven recombination and *Grin1*-ablation were demonstrated only by 8 weeks (Carlén et al., 2011), indicating that a developmental requirement of NMDAR functions was not examined in these studies. Hence it is unsurprising that while NMDAR-ablation in *Pvalb*^*Cre*^ lines produces other behavioral deficits unrelated to the Sz-like phenotypes (Korotkova et al., 2010; Bygrave et al., 2016), a developmental, but not adult-onset *Grin1*-ablation in *Ppp1r2^*Cre*^* line that targets a subset of PV interneurons among other subtypes, recapitulates core Sz-like behavioral phenotypes (Belforte et al., 2010). Emerging new mouse lines that provide genetic access into PV cells earlier than *Pvalb*^*Cre*^ (Harris et al., 2014; Wamsley et al., 2018) could address the specific requirement of developmental *Grin1* function in PV interneurons. Lastly, NMDA signaling in non-PV interneuron subtypes are also known to drive robust dendritic inhibition in pyramidal neurons (Chittajallu et al., 2017; Ali et al., 2020).

Integrating these ideas and based on findings from the present study, we propose the following: *(i)* Despite a smaller NMDAR conductance in PV interneurons, we observe a robust transcriptional coupling via NMDARs, as observed by several distinct gene expression abnormalities in this cell type relevant to human Sz. Therefore, PV-expressed NMDARs primarily serve to regulate transcriptional coupling, mediating PV-subtype abundances. *(ii)* The developmental window for NMDAR loss of function is particularly important because, its transcriptional regulation maintains the correct synaptogenic and assembly cues, which when lost, lead to disease-causing impaired connectivity. Perhaps, in the *Grin1^*fl/fl*^: Pvalb^*Cre*^* mouse line, the *Grin1*-ablation occurs only at a developmental window when synaptic connectivity is sufficiently complete, explaining why the animal model does not lead to profound Sz-like behavioral impairments (Bygrave et al., 2016). ***(iii)*** The dendrite targeting SST and NGFC interneurons also exhibit robust NMDAR signaling and transcriptional coupling. During aberrant NMDAR-transcriptional coupling, it is therefore likely that impaired dendritic connectivity and inhibition onto pyramidal neurons also contributes toward disease etiology. Therefore, our dataset provides credence to interneuronal subtype-specific granularity, connectivity and excitability, all playing combinatorial and mutually supporting roles during disease etiology. Taken together, our study presents a rich resource, laying the road map for systematic examination of NMDAR signaling in interneuron subtypes, by providing multiple molecular targets for examination in both normal and impaired circuits.

## Materials and Methods

### Contact for Reagent and Resource Sharing

Further information and requests for resources and reagents should be directed to and reasonable requests will be fulfilled by the Lead Contact, Chris J. McBain.^1^

### Animals

All experiments were conducted in accordance with animal protocols approved by the National Institutes of Health. MGE-derived interneuron-specific *Grin1^*fl/fl*^* line, and Ribotag crosses were conducted as indicated in Figure 1-Supplement 1A and Figure 6-Supplement 1E. Littermate MGE-*Grin1^*wt/wt*^* controls, and MGE-*Grin1^*fl/fl*^* male and female mice were used during this study. Mice were housed and bred in conventional vivarium with standard laboratory chow and water in standard animal cages under a 12hr circadian cycle. Genotyping of the mice were performed as indicated in the appropriate Jackson Laboratory mice catalog.

### Single-Cell Dissociation and FACS

P18-20 juvenile *Nkx2-1^*Cre*^:Grin1^*wt/wt*^:*TdT^+^ and *Nkx2-1^*Cre*^*:*Grin1^*fl/fl*^*:TdT^+^ mice were used for single-cell sequencing experiments. All mice were deeply anesthetized with isoflurane and then rapidly decapitated. Brain dissection, slicing and FACS sorting were carried out as described (Tanaka et al., 2008; Harris et al., 2018; Muñoz-Manchado et al., 2018), with slight modifications. NMDG-HEPES–based solution (Tanaka et al., 2008) was used in all steps to enable better recovery of the cells during FACS sorting and single-cell bar coding. Briefly, the brain sectioning solution contained NMDG-HEPES–based high-Mg^2+^ cutting solution contained 93 mM NMDG, 2.5 mM KCl, 1.2 mM NaH_2_PO_4_, 30 mM NaHCO_3_, 20 mM HEPES, 25 mM glucose, 5 mM sodium ascorbate, 3 mM sodium pyruvate, 2 mM Thiourea, 10 mM MgSO_4_^∗^7H_2_O, and 0.5 mM CaCl_2_^∗^2H_2_O; it was adjusted to pH 7.4 with 12.1N HCl, an osmolarity of 300–310 mOsm, and carbogenated (mix of 95% O_2_ and 5% CO_2_) before use. This solution was chilled, and the process of sectioning were conducted on an ice-chamber in the vibratome.

3-4, *Nkx2-1^*Cre*^:Grin1^*wt/wt*^:*TdT^+^ or *Nkx2-1^*Cre*^*:*Grin1^*fl/fl*^*: TdT^+^ mice were processed on consecutive days for single-cell sequencing experiments. TdT negative animals were processed in parallel for initially setting FACS gate for the Tomato-channel. Across the replicates, 10X MGE-*Grin1^*wt/wt*^* and 6X MGE-*Grin1^*fl/fl*^* animals were used for the scRNAseq. Coronal slices containing frontal cortex and hippocampus (350 mM) were cut using VT-1000S vibratome (Leica Microsystems) in cold NMDG-HEPES–based high-Mg^2+^ cutting solution. Slices were recovered in the same solution at 20°C for 30 min during when, they were visually inspected under fluorescence microscope and micro dissected, all under constant carbogenation. The recovery and microdissection were conducted in the NMDG-HEPES high-Mg^2+^ solution supplemented with 0.5 μM tetrodotoxin (TTX), 50 μM DL -2-Amino-5-phosphonopentanoic acid (APV) and 10 μM Actinomycin-D (Act-D).

Cell dissociation was performed using the Worthington Papain Dissociation System according to manufacturer instructions with minor modifications. Briefly, single-cell suspensions of the micro dissected frontal cortices or hippocampus were prepared using sufficiently carbogenated dissociation solution (containing Papain, DNAse in Earle’s Balanced Salt Solution, EBSS), supplemented with 1 μM TTX, 100 μM APV, and 20 μM Act-D. After a 60 min enzymatic digestion at 37°C, followed by gentle manual trituration with fire-polished Pasteur pipettes, the cell dissociates were centrifuged at 300 g for 5 min at 20°C, and the supernatants were discarded. The enzymatic digestion was quenched in the next step by the addition of ovomucoid protease inhibitor. Albumin density gradient was performed on the pellets, using a sufficiently carbogenated debris removal solution (containing albumin-ovomucoid inhibitor, DNAse in EBSS). The resulting cell pellets were resuspended in 1 ml FACS buffer containing 10% FBS, 10 U/μl of DNAse, 1 μM TTX, 100 μM APV, and 20 μM Act-D in a 50:50 mix of carbogenated EBSS: NMDG-HEPES–based cutting saline (with 1 mM MgSO_4_^∗^7H_2_O, it is important to not use High-Mg^2+^ in the FACS buffer, as it interferes with the subsequent 10X scRNAseq reaction). Cells were placed in polystyrene tubes (Falcon 352235) on ice during the FACS.

For single cell sorting of TdT^+^ expressing cells by FACS, resuspended cell dissociates were filtered through 35 mm cell strainer (Falcon 352235) to remove cell clumps. The single cell suspensions were then incubated with 1 μg/ml DAPI and 1 μM DRAQ5 at 4°C for 5 min to label dead cells and live cells, respectively. Samples were analyzed for TdTomato expression and sorted using a MoFlo Astrios EQ high speed cell sorter (Beckman Coulter). TdT-negative cells were used as a control to set the thresholding FACS gate for the detection and sorting of the Ai14-TdTomato-expressing cells, and the same gate was then applied for all subsequent experiments. Flow data analysis and setting of sorting gates on live (DAPI-negative, DRAQ5-positive) and Ai14-TdTomato-expressing cells were carried out using Summit software V6.3.016900 (Beckman Coulter). Per sample/session, 20,000–40,000 individual cells were sorted into a FBS-precoated, Eppendorf LoBind Microcentrifuge tubes containing carbogenated 10 ml FACS buffer, that served as the starting material for 10X Genomics barcoding.

### 10X Genomics Chromium

The cells were inspected for viability, counted, and loaded on the 10X Genomics Chromium system, aiming to recover ∼5,000 cells per condition. 12 PCR cycles were conducted for cDNA amplification, and the subsequent library preparation and sequencing were carried out in accordance with the manufacturer recommendation (Chromium^TM^ Single Cell 3′ Library and Gel Bead Kit v2 and v3, 16 reactions). Sequencing of the libraries were performed on the Illumina HiSeq 2500 at the NICHD, Molecular Genomics Core facility. **Replicate 1** of the scRNAseq were performed using 10X v2 reaction from which, the cell estimates, mean reads per cell (raw), median genes per cell, respectively, are as follows Cortical WT: 1277, 149K, 4615; Cortical NULL: 181, 159K, 4826; Hippocampal WT: 2221, 92K, 2578; Hippocampal NULL: 404, 154K, 4903. **Replicate 2** of the scRNAseq were performed using 10X v3 reaction from which, the cell estimates, mean reads per cell (raw), median genes per cell, respectively, are as follows Cortical WT: 3851, 22.8K, 1536; Cortical NULL: 2898, 23.5K, 2759; Hippocampal WT: 4600, 23.6K, 850; Hippocampal NULL: 4436, 25.8K, 3143. Replicate 3 of the scRNAseq were performed using 10X v3 reaction from which, cell estimates, mean reads per cell (raw), median genes per cell respectively, are as follows Cortical WT: 3960, 24.8K, 2870; Hippocampal WT: 3159, 26.9K, 2956. Representative quality metrics from Replicate 2 are indicated in Figure 1-Supplements 1B–E. Demultiplexed samples were aligned to the mouse reference genome (mm10). The end definitions of genes were extended 4 k bp downstream (or halfway to the next feature if closer) and converted to mRNA counts using the Cell Ranger Version 2.1.1, provided by the manufacturer.

### MGE-Ribotag-Seq

This assay was performed as recently described (Sanz et al., 2009; Mahadevan et al., 2020). Notably, *Nkx2.1^*cre*^: Grin1^*fl/wt*^* were bred with Ribotag mice for > 10 generations to obtain homozygosity in *Rpl22^*HA/HA*^*. Age-matched *Nkx2-1^*Cre*^: Grin1^*wt/wt*^: Rpl22^*HA/HA*^* or *Nkx2-1^*Cre*^*: *Grin1^*fl/fl*^*: *Rpl22^*HA/HA*^* littermates (2 males and 2 females per genotype) were utilized for the MGE-Ribotag-seq. RNAs bound with anti-HA immunoprecipitates and RNA from bulk tissue (input) were purified using RNeasy Plus Micro Kit and the quality of RNA were measured using RNA 6000 Pico kit and 2100 Bioanalyzer system. cDNA libraries were constructed from 250 pg RNA using the SMARTer Stranded Total RNA-Seq. Kit v2 only from samples with RNA Integrity Numbers > 6. Sequencing of the libraries were performed on the Illumina HiSeq2500, at 50 million 2 × 100 bp paired-end reads per sample. ∼75% of reads were uniquely mapped to genomic features in the reference genome. Bioconductor package DESeq2 (Love et al., 2014) was used to identify differentially expressed genes (DEG). This package allows for statistical determination of DEGs using a negative binomial distribution model. The resulting values were then adjusted using the Benjamini and Hochberg’s method for controlling the false discovery rate (FDR).

### Data Processing, Analyses, Visualization, and Differential Expression Testing

Processing (load, align, merge, cluster, differential expression testing) and visualization of the scRNAseq datasets were performed with the R statistical programming environment (R Core Team, 2013) (v3.5.1) and Seurat package (Butler et al., 2018; Stuart et al., 2019) (v3.1.5, a development version of Seurat v3.1.5.9000 was used to generate violin plots in Figures 2C, 7B, Figure 7-Supplement 1, and Figure 2-Supplement 4A). Data set preprocessing, comparison of WT- and NULL-Ai14 cells, canonical correlation analyses, and differential expression of genes (padj < 0.01) within the same cluster between WT- and NULL-Ai14 cells were performed according to default Seurat parameters, unless otherwise mentioned. Quality control filtering was performed by only including cells that had between 200 and 6,000 unique genes, and that had < 30% of reads from mitochondrial genes. While the WT replicates had no cells above 30% mitochondrial genes, only NULL replicates from both brain regions exhibited 7–12% of cells above this threshold. Suggestive of inherent biological impact of *Grin1*-ablation, we repeated the clustering and subsequent analyses without excluding any cells. These analyses did not alter the clustering or skew the gene list. Clustering was performed on the top 25 PCs using the function FindClusters() by applying the shared nearest neighbor modularity optimization with varying clustering resolution. A cluster resolution of 1.0 was determined to be biologically meaningful, that yielded all known MGE cardinal classes. Initial analyses were performed on the WT datasets separately (WT.alone), and similar set of analysis parameters were applied when the WT and NULL samples were merged (WT.NULL.integrated) for subsequent differential expression testing. Phylogenetic tree relating the “average” cell from each identity class based on a distance matrix constructed in gene expression space using the BuildClusterTree() function. Overall, we identified 27, and 33 clusters using this approach in the WT.alone, and WT.NULL.integrated assays, respectively. The WT.alone correspond to 11 MGE.*Gad1/Gad2* clusters (Figure 1C), while the WT.NULL.integrated assay correspond to 12 clusters (Figure 2B). We first searched for the top differential markers for each MGE subcluster using the FindAllMarkers() function. The genes thus identified for the integrated data is presented in Supplementary Table 1B. Determination of MGE and non-MGE identities are performed based on existing interneuron literature and other scRNAseq datasets (Tasic et al., 2016, 2018; Paul et al., 2017; Pelkey et al., 2017; Harris et al., 2018; Fishell and Kepecs, 2020). The labels from Figures 1, 2 are matched with the top gene markers identified by the FindAllMarkers() function and the similarly named clusters in Figures 1, 2 have the same identities. Lastly, for the integrated analyses and differential expression testing, we first merged the identities of the subclusters SST.1-SST.6 and TH.1 and relabeled as SST subset; PVALB.1-3 relabeled as PVALB subset; and NGFC.1-2 relabeled as the NGFC subset during subsequent analysis (Figures 5–8).

Differential gene expression testing was performed using the MAST package (Finak et al., 2015) within the FindMarkers function to identify the differentially expressed genes between two subclusters. MAST utilizes a hurdle model with normalized UMI as covariate to generate the differential fold changes and is known to result in underestimation of the magnitude of fold change (FC) (Ximerakis et al., 2019). Therefore, while applying a stringent false-discovery rate < 0.01, we determined the minimum FC based on the control gene *Grin1*, which is the target gene knocked out in MGE-derived interneuron cell types. Notably for *Grin1*, we had previously demonstrated that the NGFCs which carry maximum NMDAR component among MGEs, are devoid of NMDAR current at this comparable age (Chittajallu et al., 2017). In the present scRNAseq assay, we observe a logFC for *Grin1* ranging between −0.1 and −0.35 across both brain regions and all MGE subtypes. Therefore, we determined a minimum logFC in our DEGs as ± 0.1 to be meaningful. Previous studies have demonstrated the MAST approach for DEG testing to be powerful in determining subtle changes in highly transcribed genes, and among abundant populations, additional to underrepresenting changes among weakly transcribed genes (Finak et al., 2015; Ximerakis et al., 2019). Volcano plots and Heat maps for the DEG were generated using EnhancedVolcano package (Blighe et al., 2021) and Morpheus package (Broad Institute) within the R framework. UpSet plots were generated using the Intervene package s within the R framework. Associated scRNAseq source data, including sequencing reads and single cell expression matrices, is available from the Gene Expression Omnibus (GEO) under accession number GSE156201.

### Pathway Analyses PPI Network Mapping and Disease Mapping

Ingenuity Pathway Analyses were conducted on the differentially expressed genes to generate the molecular functional annotation and to identify the biological pathways and disease pathways overrepresented. This tool was also used to annotate genes with their known cellular functional classes. Additional Gene Ontology mapping and KEGG analyses were conducted using ShinyGO (Ge et al., 2020). Protein-protein interaction (PPI) mapping datasets from a variety of curated databases were conducted as previously described (Mahadevan et al., 2017) using the Cytoscape platform (v3.8.0) (Smoot et al., 2011). Schizophrenia risk genes integrated from various sources including genome-wide association studies (GWAS), copy number variation (CNV), association and linkage studies, post-mortem human brain gene expression, expression quantitative trait loci (eQTL) and encyclopedia of DNA elements (ENCODE), were downloaded from http://www.szdb.org/ (Wu Y. et al., 2017). Autism Spectrum Disorder risk genes integrated from various sources were downloaded from Simons Foundation https://gene.sfari.org/ (Simons Foundation, 2018). SZDB genes that had an integrated total score of 3-6 (1419 genes, 22% out of 6387) were considered “high-risk” for DEG mapping (Supplementary Table 4A). SFARI genes scored 1-2 with accounting for a high strength of evidence (392 genes, 42% out of 943), were considered “high-risk” for DEG mapping (Supplementary Table 4B).

### Immunostaining

All solutions were freshly prepared and filtered using 0.22μm syringe filters for parallel treatments of wildtype and MGE-*Grin1*-null groups. Adult mice of postnatal day (PD) 25–30 were mice were deeply anesthetized with isoflurane and perfused transcardially with 1X phosphate buffer saline (PBS) and followed by the fixative 4% paraformaldehyde. The brains were post-fixed in the same fixative for overnight at 4°C for the immunostaining assays. Postfixed brains were serially dehydrated using 10%/20%/30% sucrose solutions at 4°C. Coronal or saggital sections (50 μm) were cut on a freezing microtome. Immunostaining was performed on free-floating sections. Tissue sections were permeabilized and blocked in 1 × PBS + 1% bovine serum albumin + 10% normal goat serum + 0.5% Triton X-100 (Carrier PB) at room temperature for 2 h, followed by incubation in primary antibodies, listed below, diluted with 1 × PBS + 1% bovine serum albumin + 1% normal goat serum + 0.1% Triton X-100 overnight at 4°C. Tissue sections were then incubated with secondary antibodies, listed below, diluted in Carrier Solution (1:1,000), and DAPI (1:2,000) at room temperature for 1–2 h and mounted on Superfrost glass slides, and coverslipped using Mowiol mounting medium and 1.5 mm cover glasses.

### Antibodies

The following primary antibodies were used: mouse anti-PV (1:1,000; Sigma-Aldrich Cat# P3088, RRID: AB_477329), rat anti-SST (1:1,000; Millipore Cat# MAB354, RRID: AB_2255365), rabbit anti-Satb1 (1:1,000; Abcam Cat# ab109122, RRID: AB_10862207), rabbit anti-HA (Abcam, Cat#ab9110, RRID AB_307019), rabbit anti Nkx2-1 (1:500, Abcam:Cat#ab76013/Epitomics:Cat#2044-1, RRID: AB_1310784). Secondary antibodies were conjugated with Alexa Fluor dyes 488 or 633 (1:1,000; Thermo Fisher Scientific).

### RNAscope HiPlex *in situ* Hybridization

All apparatus, dissection instruments for this assay were treated to maintain RNAse-free conditions. Brains from two sets of P20-25 littermates from both genotypes were rapidly dissected and rinsed in fresh ice-cold RNAse-free 1XPBS. Individual brains were placed into cryomolds and snap-frozen by dipping into isopentene:dry ice mix. Frozen brains were stored in −80°C. 15μm fresh frozen saggital sections were obtained using a RNAse-free cryostat, and sections serially mounted on RNAse-free Superfrost glass slides. After a series of pretreatments with 4% fresh PFA and dehydration using 50, 70, and 100% EtOH, the slides were processed for HiPlex assay according to ACDBio manufacturer’s instructions within a week. A panel of HiPlex probes were utilized counterstained with DAPI (1:2,000), but only data from *Reln, Sst, Nos1* are presented in this study.

### Image Acquisition and Analysis

#### Immunostaining

Mouse brains from 3 to 6 animals (2–4 sections per animal) were used for each condition, and section depth were matched between the genotypes for parallel immunostaining. Fluorescent images were captured using the 20X objective of a Nikon Widefield Fluorescence, Spinning Disk Confocal microscope. For all slices with immunostained or genetically reported signal, 50 μm thin sections were imaged using 20x/0.45 CFI Plan Apo objective (imaging settings: Numerical Aperture 0.75, bit depth 16-bit, Exposure 100 ms). 10–20 Confocal z-stacks were acquired at a depth of 2 μM, and were stitched using NIS Elements (Nikon) before importing them into Imaris software (Bitplane, version 9.6). Cell bodies were marked in Imaris software using the “Spots” function. *Nkx2.1^*Cre*^* TdT^+^, PV^+^, SST^+^, SATB1^+^, NKX2-1^+^ cell bodies were detected using the automatic function, with a signal detection radius of 15 μm. The Imaris “Quality” filter was set above an empirically determined threshold to maximize the number of detected cells while minimizing observed false positives. SST + cell bodies were marked manually using the Imaris “Spots” function. ROI 3D borders around hippocampus or somatosensory cortex, drawn manually using the Imaris function “Surfaces.” Spots were then split within each ROI using the Imaris function “Split Spots.” Overlap of TdT^+^ cells with other markers (PV, SST, SATB1, NKX2-1) was addressed by filtering the TdT^+^ Spots above an empirically determined threshold intensity in the channel relative to the marker of interest. Each image with an automatic analysis by Imaris was checked by an expert and incorrectly identified cell bodies where refined if required. Total numbers of cell counts are normalized to the volume of the section imaged and normalized to the TdT cell numbers where applicable. In Figure 2-Supplement 1, Figure 4, Figure 4-Supplement 1, and Figure 6E, error bars reflect standard error of mean (SEM); Two-tailed unpaired *t*-test was performed using Prism8.

#### RNAscope-HiPlex

Mouse brains from 2 animals (3–5 sections per animal) were used for each condition, and section depth were matched between the genotypes for parallel HiPlex assay. Fluorescent images were captured using the 40X-oil objective of a Nikon Widefield Fluorescence, Spinning Disk Confocal microscope. All sections were imaged using 10x/0.45 CFI Plan Apo objective (imaging settings: Numerical Aperture 0.75, bit depth 16-bit, Exposure 100 ms). Confocal stacks were stitched using NIS Elements (Nikon) before importing them into Imaris software (Bit-plane, version 9.6). *Sst* + *Reln* + *Nos1* + RNA signals that contain DAPI were identified using “Spot” function as described above, and the overlap of *Sst* + spots with *Reln* + or *Nos1* + spots were identified by thresholding the signals of *Reln* or *Nos1*, respectively. Total numbers of cell counts are normalized to the area of the thin section imaged. In Figure 3, error bars reflect standard error of mean (SEM); Two-tailed unpaired *t*-test was performed using Prism8.

## Data Availability Statement

scRNAseq source data are available from the Gene Expression Omnibus (GEO) under accession number GSE156201 (https:// www.ncbi.nlm.nih.gov/geo/query/acc.cgi?acc=GSE156201).

## Ethics Statement

All experiments were conducted in accordance with animal protocols approved by the National Institutes of Health.

## Author Contributions

VM and CJM conceived the project and wrote the manuscript. VM, TJP, RC, KAP, and CJM designed the experiments. DM performed FACS sorting and analysis. VM, YZ, and TJP performed 10X scRNAseq. VM performed Ribotag assays. VM, AM, CR, CE, and RD performed 10X scRNAseq bioinformatic analyses. VM, XY, and KAP conducted RNAscope, immunofluorescent staining, imaging, and analysis. CJM supervised the study. All authors edited the manuscript.

## Conflict of Interest

The authors declare that the research was conducted in the absence of any commercial or financial relationships that could be construed as a potential conflict of interest.

## Publisher’s Note

All claims expressed in this article are solely those of the authors and do not necessarily represent those of their affiliated organizations, or those of the publisher, the editors and the reviewers. Any product that may be evaluated in this article, or claim that may be made by its manufacturer, is not guaranteed or endorsed by the publisher.

## Footnotes

1
mcbainc@mail.nih.gov
