# Supplementary material for: NMDARs Drive the Expression of Neuropsychiatric Disorder Risk Genes Within GABAergic Interneuron Subtypes in the Juvenile Brain
Source: Front Mol Neurosci. 2021 Sep 14;14:712609. doi: 10.3389/fnmol.2021.712609 (PMC8500094; doi:10.3389/fnmol.2021.712609)
Supplement: Supplementary file 1 [file Presentation_1.pdf]

# NMDARs drive the expression of neuropsychiatric disorder risk genes within GABAergic interneuron subtypes in the juvenile brain

Vivek Mahadevan1*, Apratim Mitra[2], Yajun Zhang[3], Xiaoqing Yuan[1], Areg Peltekian[1], Ramesh Chittajallu[1], Caroline Esnault[2], Dragan Maric[4], Christopher Rhodes[3], Kenneth A. Pelkey[1], Ryan Dale[2†,] Timothy J. Petros[3‡] & Chris J. McBain[1§¶]

[1]Section on Cellular and Synaptic Physiology, Eunice Kennedy Shriver National Institute of Child Health and Human Development (NICHD), Bethesda, 20892, MD, USA
[2]Bioinformatics and Scientific Programming Core, NICHD, Bethesda, 20892, MD, USA
[3]Unit on Cellular and Molecular Neurodevelopment, NICHD, Bethesda, 20892, MD, USA
[4]Flow and Imaging Cytometry Core Facility, National Institute of Neurological Disorders and Stroke (NINDS), Bethesda, 20852, MD, USA.

*E-mail: vivek.mahadevan@nih.gov
†E-mail: ryan.dale@nih.gov
‡E-mail: tim.petros@nih.gov

§Corresponding author:
¶E-mail: mcbainc@mail.nih.gov

# Supplementary Figure Legends

## FIGURE1, SUPP1 | Schematic overview and quality control for scRNAseq

**A,** Breeding strategy, **$B_i$, $C_i$, $D_i$, $E_i$**, Representative FACS gates to sequentially isolate live: dead cells using DAPI: DRAQ5 staining, singlet-gating and TdT$^+$-reporter gating to obtain reporter-positive MGE-derived interneurons from frontal neocortex and hippocampus. **$B_{ii}$, $C_{ii}$, $D_{ii}$, $E_{ii}$**, Barcode Rank Plots for cells from WT and NULL mice, demonstrating separation of cell-associated barcodes and those associated with empty partitions. UMI, unique molecular identifier; MR, Mean Reads; MG, Median Genes. **$B_{iii}$, $C_{iii}$, $D_{iii}$, $E_{iii}$**, Distributions of the total number of genes, percentage of mitochondrial genes and UMIs per cell in control mice. **$B_{iv}$, $C_{iv}$, $D_{iv}$, $E_{iv}$,** Pearson correlation coefficient of the distributions of the total number of genes and the UMI

## FIGURE1, SUPP2 | Biological replicates

Representative UMAP plots of the 3 biological replicates from neocortex and hippocampus indicates **A,** similar clustering, and **B,** similar expression profiles of *Nkx2.1*-derived, *Gad1*-expressing MGE-derived interneurons and *Nkx2.1* derived, *Olig1*-expressing oligodendrocytes.

## FIGURE1, SUPP3 | Select marker gene expression across the subtypes of merged cortical and hippocampal MGE-*Grin1*$^{wt/wt}$

**A$_{i}$,** UMAP representation of cardinal MGE markers genes in the cortical and hippocampal merged dataset. **A$_{ii}$,** UMAP representation colored by region, highlighting the region-specific enrichments of MGE subsets. **A$_{iii}$,** Pie chart indicating the percentages of cells recovered across the interneuron subtypes from neocortex and the hippocampus. **B,** Violin plot showing the distribution of expression levels of well-known representative cell-type-enriched marker genes across the 11 MGE subtypes.

**FIGURE1, SUPP4 | MGE-derived interneuron subtype differences between neocortex and hippocampus**

Volcano plot representing the −log10 False Discovery Rate (FDR) versus log2 fold change (FC) between **A,** TH-expressing MGE subsets and the remaining MGE subset SST, PVALB and NGFC; **B,** Differential expression of the cardinal MGE classes between neocortex and hippocampus, at a fold change ≥0.5 and FDR <10e-5.

## FIGURE1, SUPP5 | MGE-derived interneuron subtype annotation based on marker expression

**A,** Table indicating the subtype-defining marker genes observed in the present study and their descriptions in the previous scRNAseq datasets (*indicates the genes expressed in the cortex-exclusive PVALB.2 subcluster). Representative UMAP plots of MGE subtype-enriched genes in **B,** SST subclusters, **C,** NGFC subclusters, and **D,** PVALB subclusters.

## FIGURE2, SUPP1 | Validation of MGE subtype abundances subsequent to *Grin1*-ablation by immunostaining

**A,** Boxplots indicating the cell counts of MGE-derived interneurons expressing **($A_i$)** Ai14/tdTomato, **($A_{ii}$)** PV, SST immunostaining from P30 somatosensory neocortex of *Grin1*$^{wt/wt}$ and *Grin1*$^{fl/fl}$. **B,** Boxplots indicating the cell counts of hippocampal MGE-derived interneurons expressing **($B_i$)** Ai14/tdTomato, and **($B_{ii}$)** PV, SST immunostaining from P30 *Grin1*$^{wt/wt}$ and *Grin1*$^{fl/fl}$.

## FIGURE2, SUPP2 | scRNAseq differential recoveries of MGE-derived interneuron subtypes

**A$_i$,** Number of cells recovered across cardinal subtypes SST, PV and NGFC. **A$_{ii}$,** Number of cells recovered within the subtypes of PV / SST/ NGFC. **B,** UMAP representation colored by cardinal MGE-derived interneuron subtypes SST, PVALB and NGFC, highlighting the differential enrichments of cells **C,** Representative UMAP plots indicating the granularity among PV/SST/NGFC subtypes between both brain regions and both genotypes.

## FIGURE2, SUPP3 | Select marker gene expression across the subtypes of merged MGE-derived interneurons from *Grin1*$^{wt/wt}$ and *Grin1*$^{fl/fl}$

**A,** Violin plot showing the distribution of expression levels of well-known representative cell-type-enriched marker genes across the MGE subtypes. **B$_i$,** −log10 False Discovery Rate (FDR) versus log2 fold change (FC) between *Pthlh*-PVALB.2 and *Pthlh*-PVALB.3 at a fold change ≥0.5 and FDR <10e-3. **B$_{ii}$,** Dot plots representing the normalized expressions of NGFC marker genes mis expressed in *Pthlh*-PVALB.3 upon *Grin1*-ablation.

## FIGURE2, SUPP4 | Expression of control genes in the MGE-derived interneuron subtypes subsequent to *Grin1*-ablation

**A,** Split-violin plot from both genotypes indicating the expression of *Grin1* in the MGE-derived interneurons, pyramidal neurons and non-neurons. **B,** Representative UMAP plots of cardinal MGE markers genes.

**FIGURE4, SUPP1 | Validation of Nkx2.1-expressing interneuron in P25-30 mouse cortex and hippocampus; anti-Nkx2-1 and anti-PV/SST co-immunostaining**

$\mathbf{A_i}$, Representative saggital section from P25-30 MGE-*Grin1*$^{wt/wt}$-mice immunostained with anti-Nkx2-1 (cyan), and counter-stained with DAPI (blue). While arrow heads in the cortex indicate Nkx2-1 (+) cells in deep and in mid-cortical layers. $\mathbf{A_{ii}}$, Boxplots indicate the normalized density of cortical vs hippocampal Ai14(+): Nkx2-1(+) double-positive cell counts normalized to total Ai14(+) counts. Representative immunostaining of P25-30 MGE-*Grin1*$^{wt/wt}$ brains from **B**, somatosensory cortex or $\mathbf{C_i}$,hippocampus using anti-Nkx2-1 (cyan), DAPI (blue), PV/SST (green) and endogenous Ai14 reporter (red) expression. $\mathbf{C_{ii}}$, Indicates rare representative example of rare PV(+): Nkx2-1(+) double-positive cells from MGE-*Grin1*$^{fl/fl}$ hippocampus. (0 cells from WT, 4 cells from null tissue; n = 3 brains (4 sections) from each genotype for immunostaining); Error bars reflect s.e.m from individual sections; two-tailed unpaired t-test, for statistical analysis.

**FIGURE5, SUPP1 | Differential gene expression in the MGE-derived interneuron subtypes subsequent to *Grin1*-ablation**

**$A_i$,** Bar plot denoting the number of genes up/downregulated in the cortical and hippocampal MGE clusters. Volcano plot representing the −log10 False Discovery Rate (FDR) versus log2 fold change (FC) between **B,** hippocampal and **C,** cortical MGE cardinal clusters upon *Grin1*-loss, at a fold change ≥0.2 and FDR <10e-6.

## FIGURE5, SUPP2 | Molecular pathways differentially expressed in MGE-derived interneuron subtypes subsequent to *Grin1*-ablation

**A,** Hierarchical clustering tree summarizing the correlation among significant pathways enriched among the DEGs. Pathways with many shared genes are clustered together. Bigger dots indicate more significant P-values. **B,** Bar plot showing the classification of molecular functions of the DEGs, across the MGE subtypes. Total number of DEGs in the particular molecular class indicated in parentheses.

## FIGURE6, SUPP1 | Differential expression of intracellular signaling cascades across subtypes upon *Grin1*-ablation

Heatmap of log2 FC of significant DEGs in cortical and hippocampal MGE cardinal subtypes, showing a subset of **A,** genes regulating intracellular $Ca^{2+}$ homeostasis and $Ca^{2+}$ binding proteins; **B,** notable second messengers; **C,** $Ca^{2+}$ dependent / activated kinases and phosphatases.and, **D,** cellular energetics and mitochondrial function. **E,** Breeding strategy to obtain MGE-derived interneuron-specific expression of Ribotag (*Rpl22-$^{HA}$*) transgene in the background of *Grin1$^{fl/fl}$* alleles.

**FIGURE7, SUPP1 | Differential expression of fundamental interneuron marker genes across subtypes upon *Grin1*-ablation**

Differential expression of cardinal interneuron marker genes that are broadly expressed across interneurons by **$A_i$,** scRNAseq approach, and cross-validated using **$A_{ii}$,** the Ribotag-seq approach.

**FIGURE8, SUPP1 | Aberrant NMDAR-signaling result in misexpression of regulators of membrane excitability that are high-risk Sz genes**

**A,** Schema representing the field of hippocampal pyramidal cell innervated by the interneurons. Heatmap of log2 FC of significant DEGs in cortical and hippocampal MGE cardinal subtypes, showing a subset of **$B_i$,** Neurotransmitter release machinery. **$B_{ii}$,** Postsynaptic GABA / Glutamate receptor complexes, Kcn-, Scn-ion channel complexes and other miscellaneous regulators of excitability.

# List of Supplementary Tables

**Table1A |** List of differentially expressed genes in MGE-interneuron subtypes after *Grin1*-ablation.

**Table1B |** scRNAseq cell number recoveries across different clusters from both *Grin1*$^{wt/wt}$ *and Grin1*$^{fl/fl}$.

**Table1C |** scRNAseq cluster marker genes from both *Grin1*$^{wt/wt}$ *and Grin1*$^{fl/fl}$.

**Table2 |** Intracellular signaling pathways differentially expressed after *Grin1*-ablation.

**Table3 |** Differentially enriched disease ontology mapping after *Grin1*-ablation.

**Table4A |** Differentially expressed genes mapped to high-ranked SZDB and SFARI databases.

**Table4B |** All schizophrenia risk genes catalogued in SZDB database.

**Table4C |** All autism risk genes catalogued in SFARI database.

**Table4D|** Protein-protein interaction statistics for disease-annotated genes
