# Supplementary figures and images for: NMDARs Drive the Expression of Neuropsychiatric Disorder Risk Genes Within GABAergic Interneuron Subtypes in the Juvenile Brain
Source: Front Mol Neurosci. 2021 Sep 14;14:712609. doi: 10.3389/fnmol.2021.712609 (PMC8500094; doi:10.3389/fnmol.2021.712609)

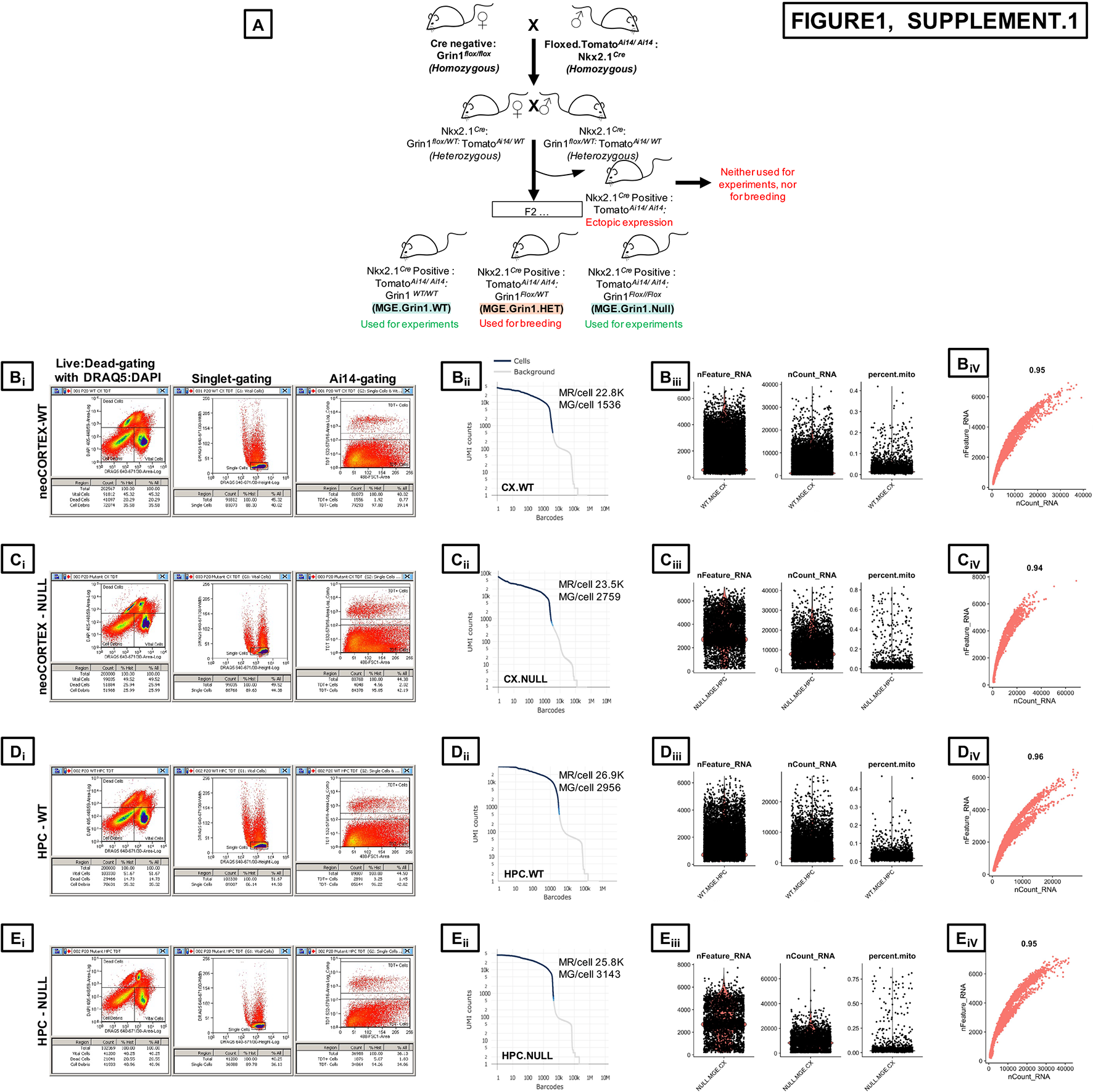

Supplement: Supplementary file 2 [file Image_1.TIF]

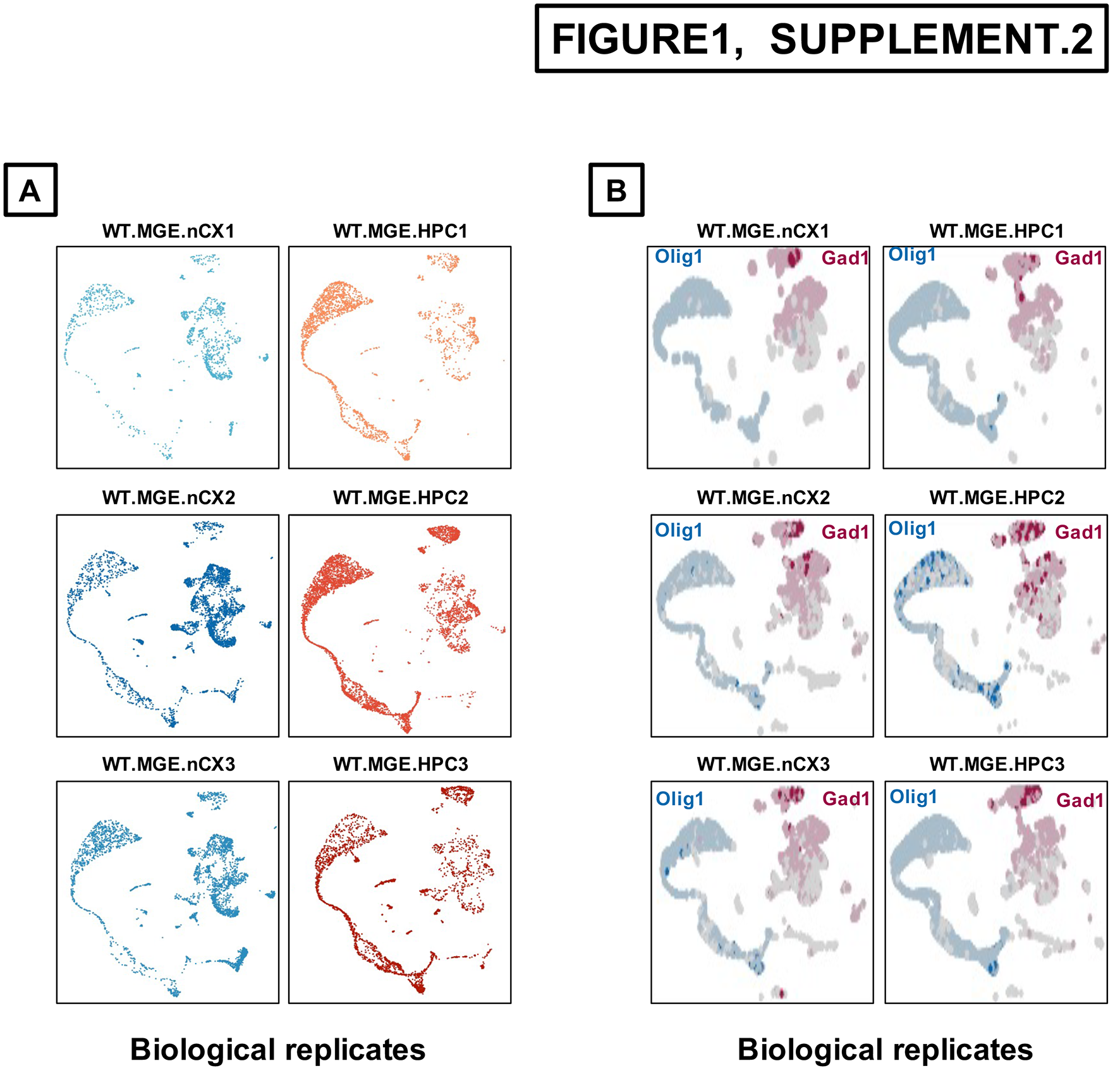

Supplement: Supplementary file 3 [file Image_2.TIF]

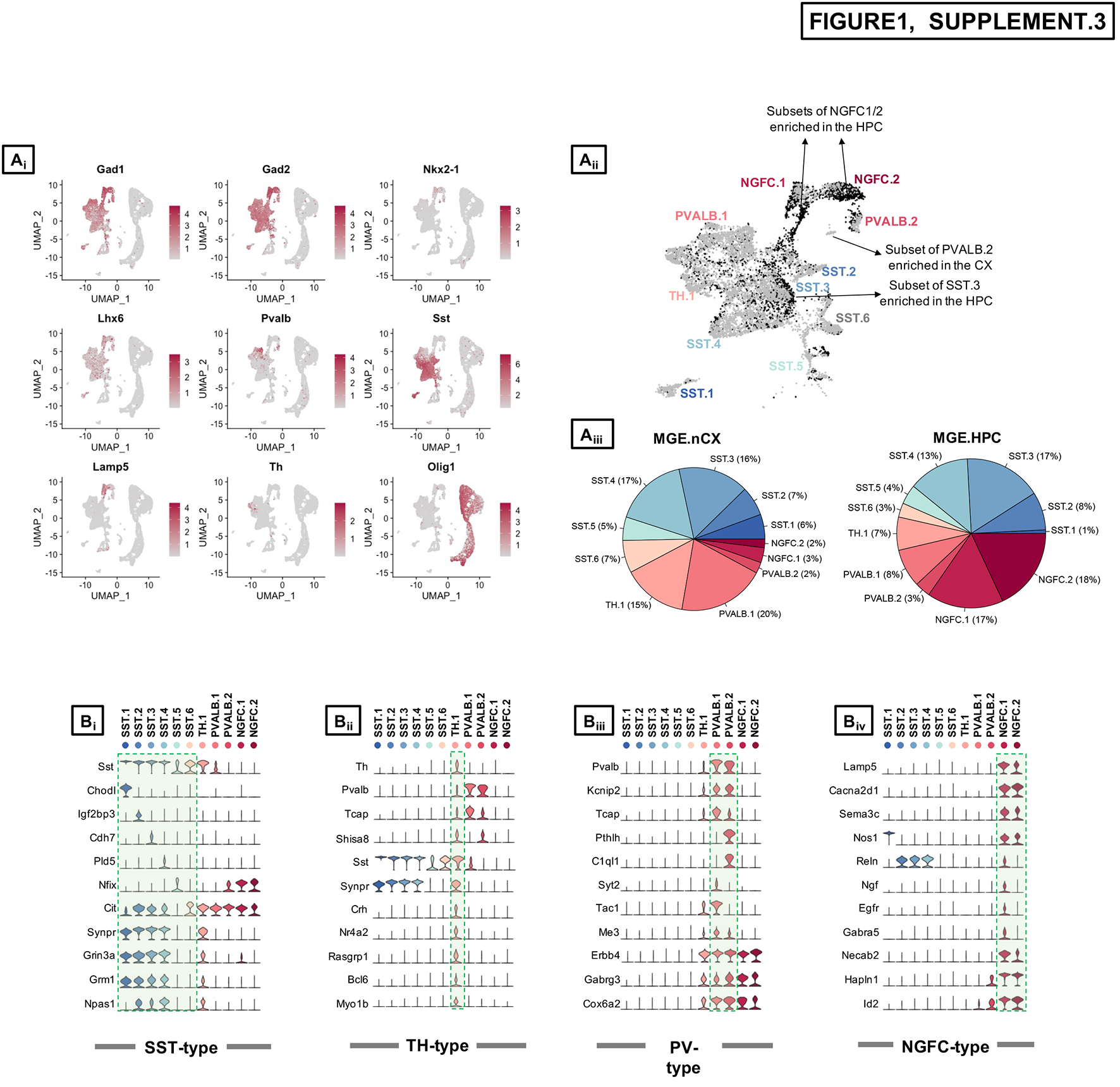

Supplement: Supplementary file 4 [file Image_3.TIF]

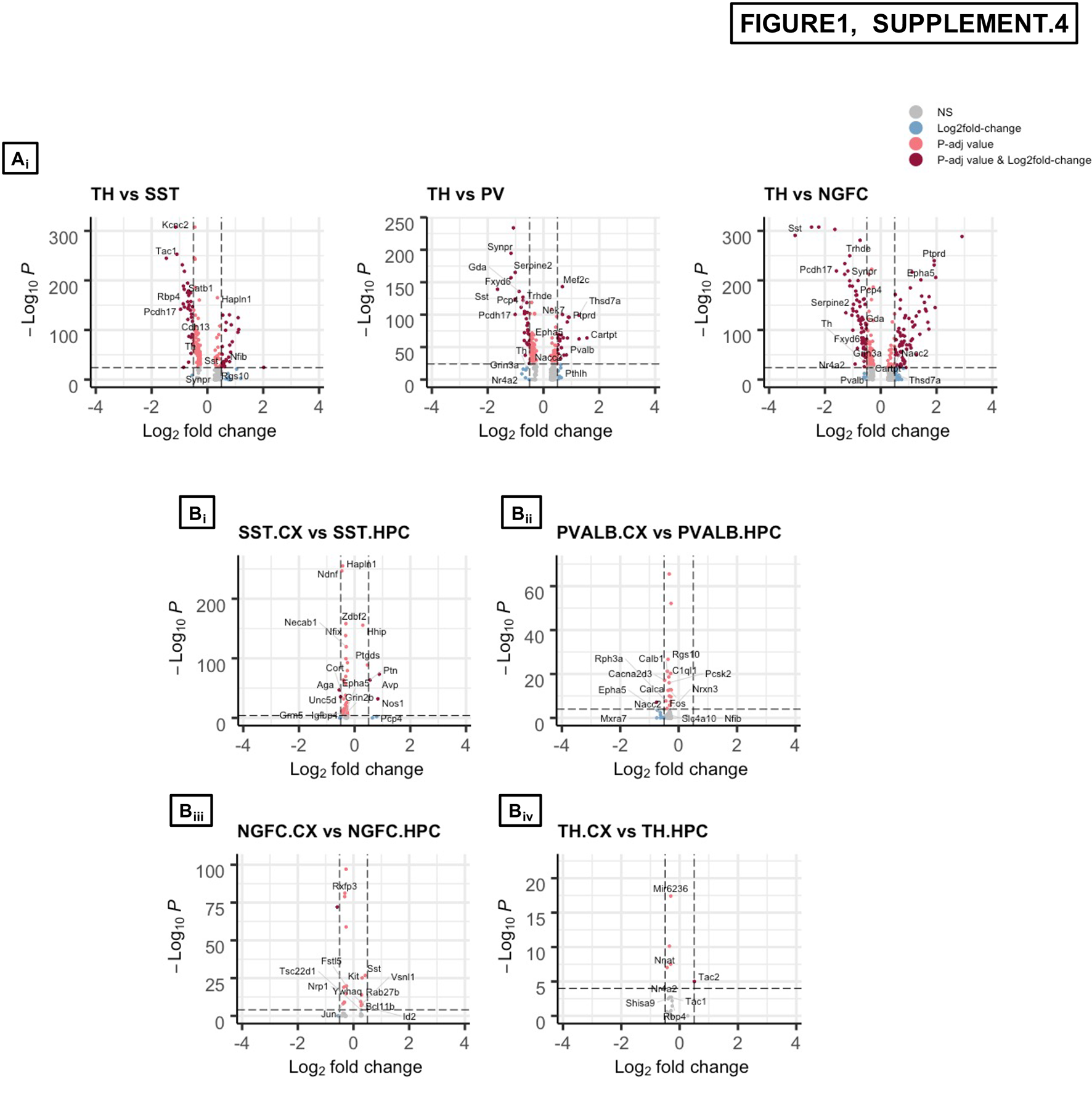

Supplement: Supplementary file 5 [file Image_4.TIF]

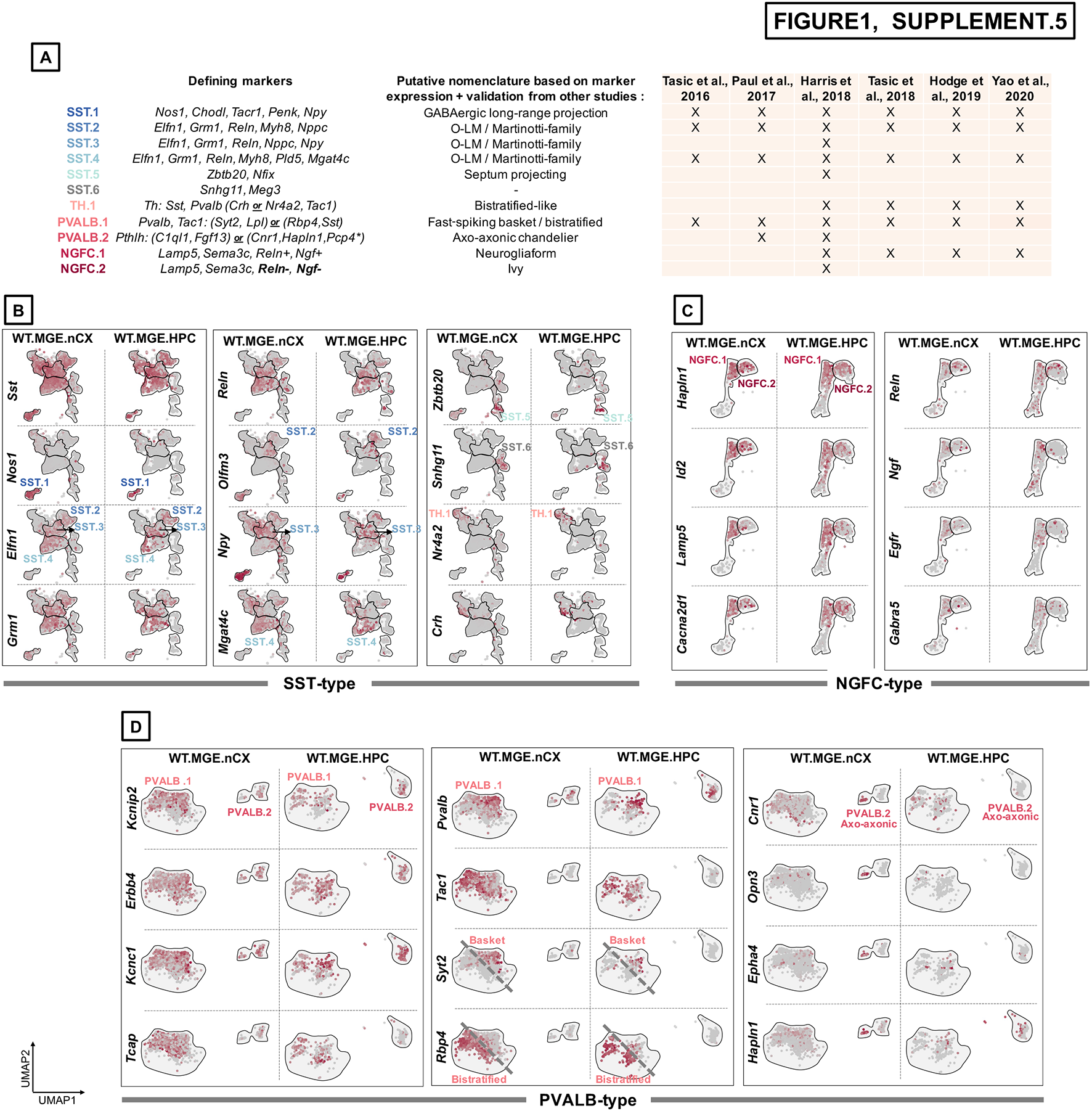

Supplement: Supplementary file 6 [file Image_5.TIF]

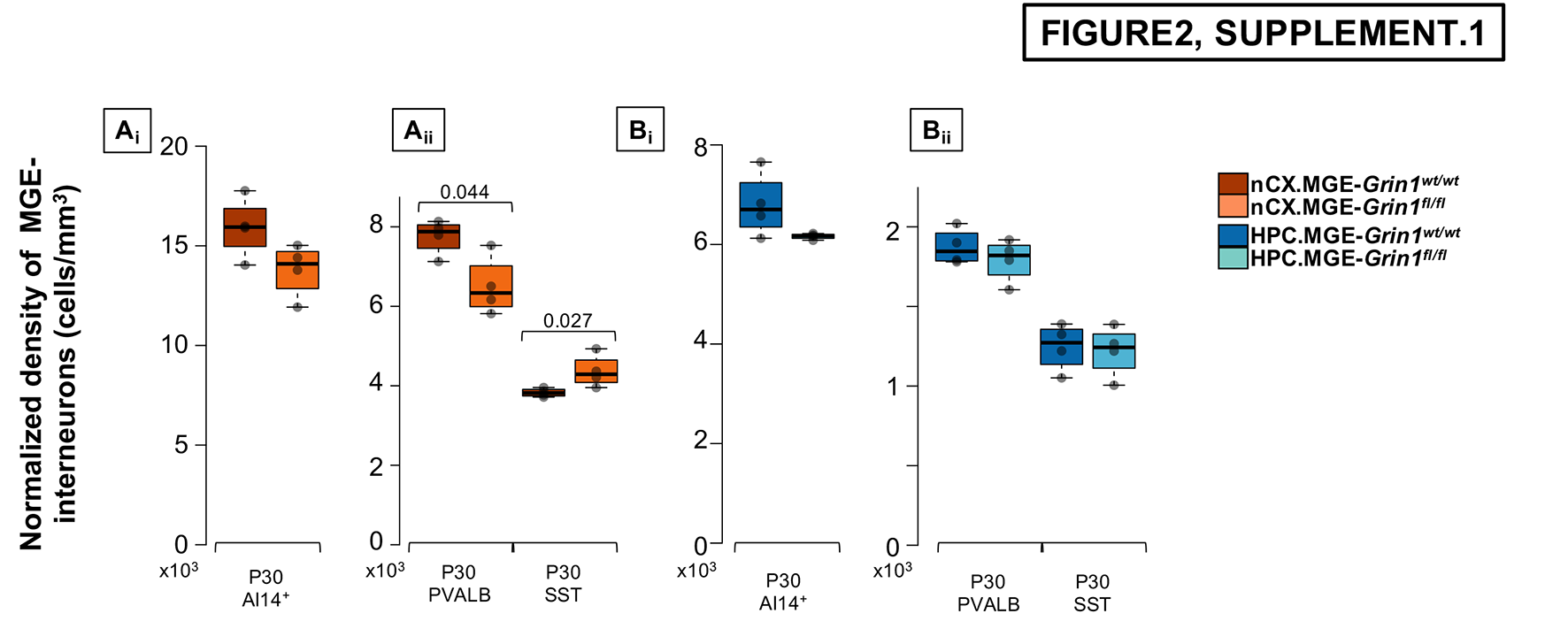

Supplement: Supplementary file 7 [file Image_6.TIF]

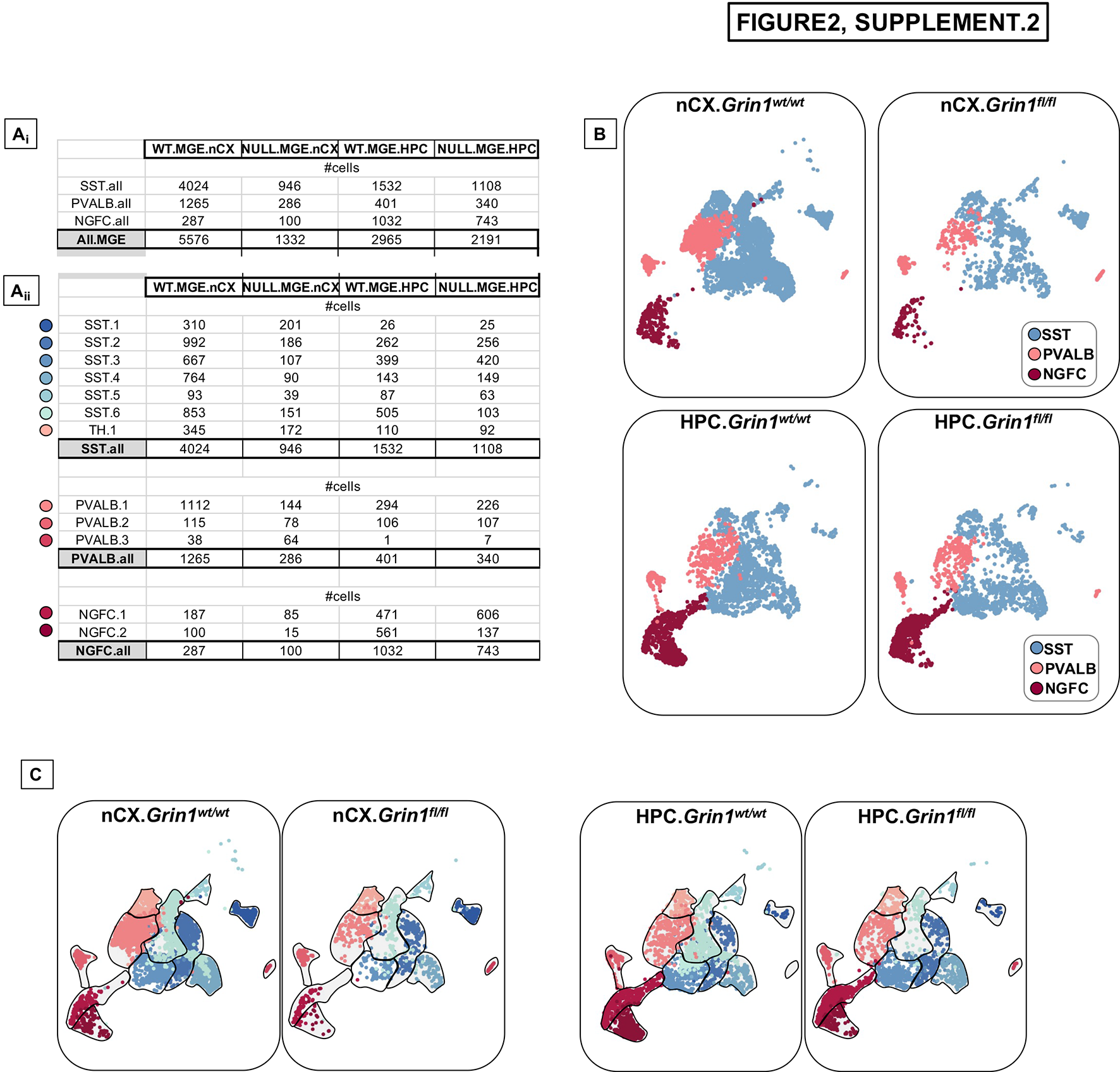

Supplement: Supplementary file 8 [file Image_7.TIF]

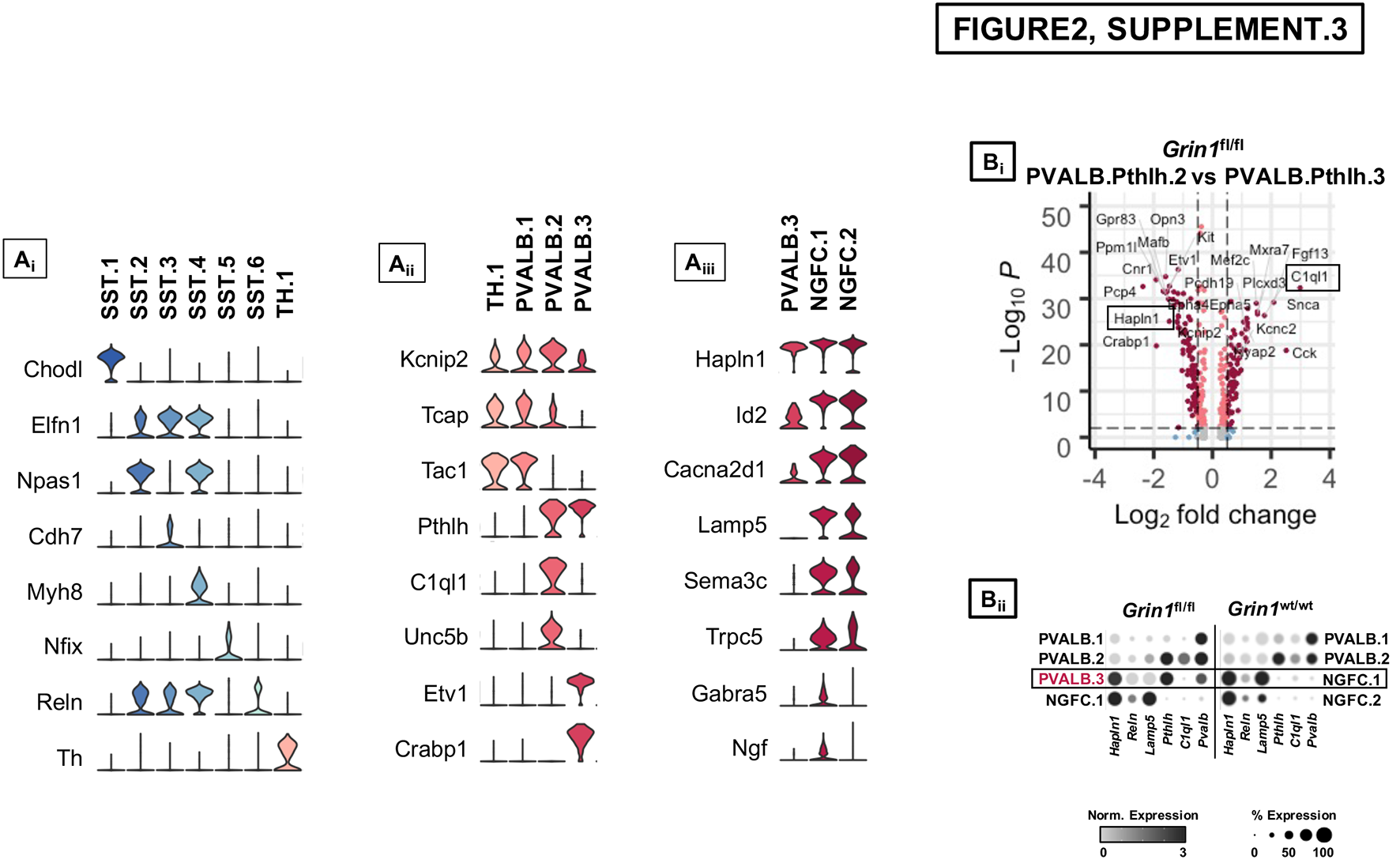

Supplement: Supplementary file 9 [file Image_8.TIF]

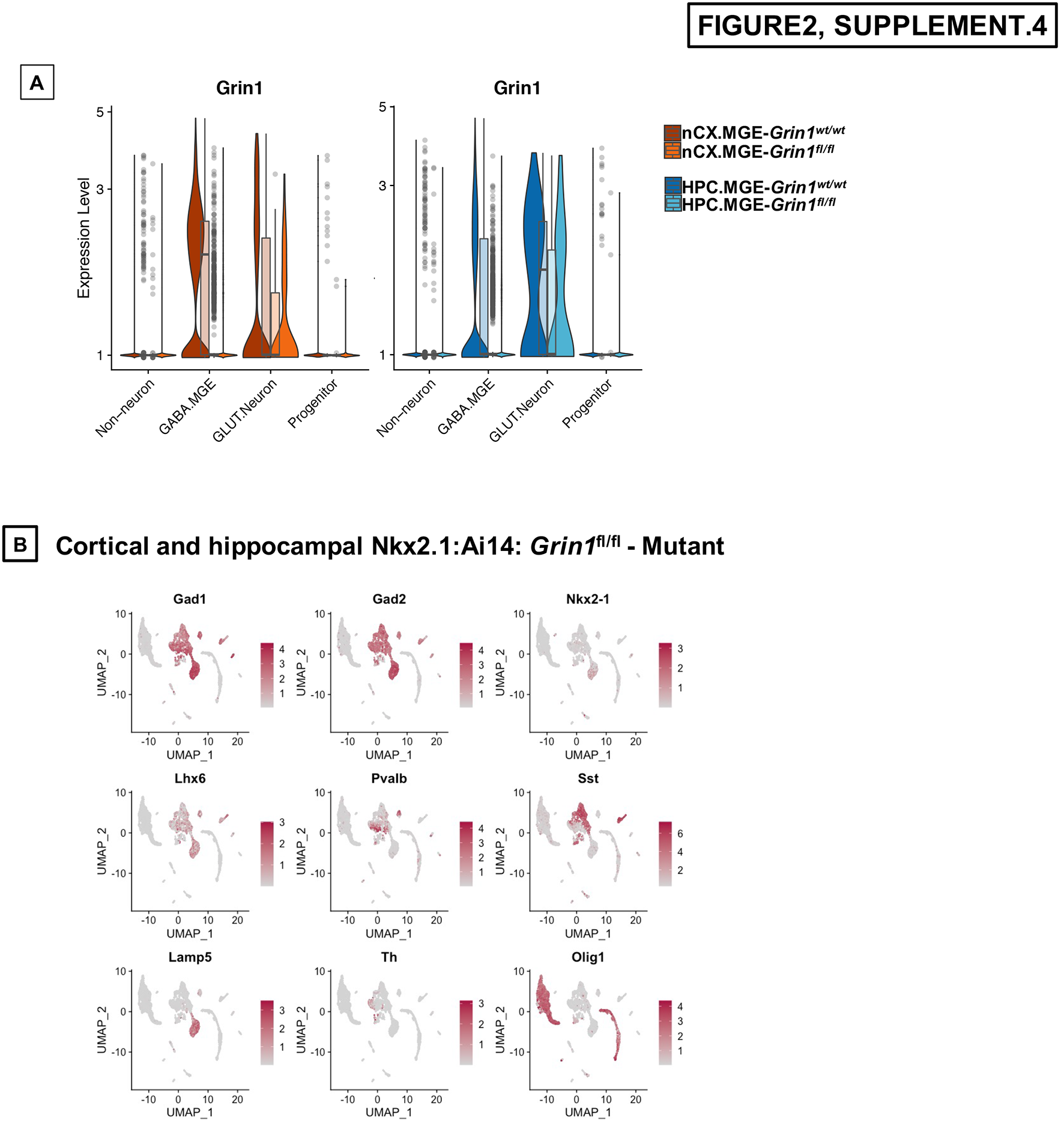

Supplement: Supplementary file 10 [file Image_9.TIF]

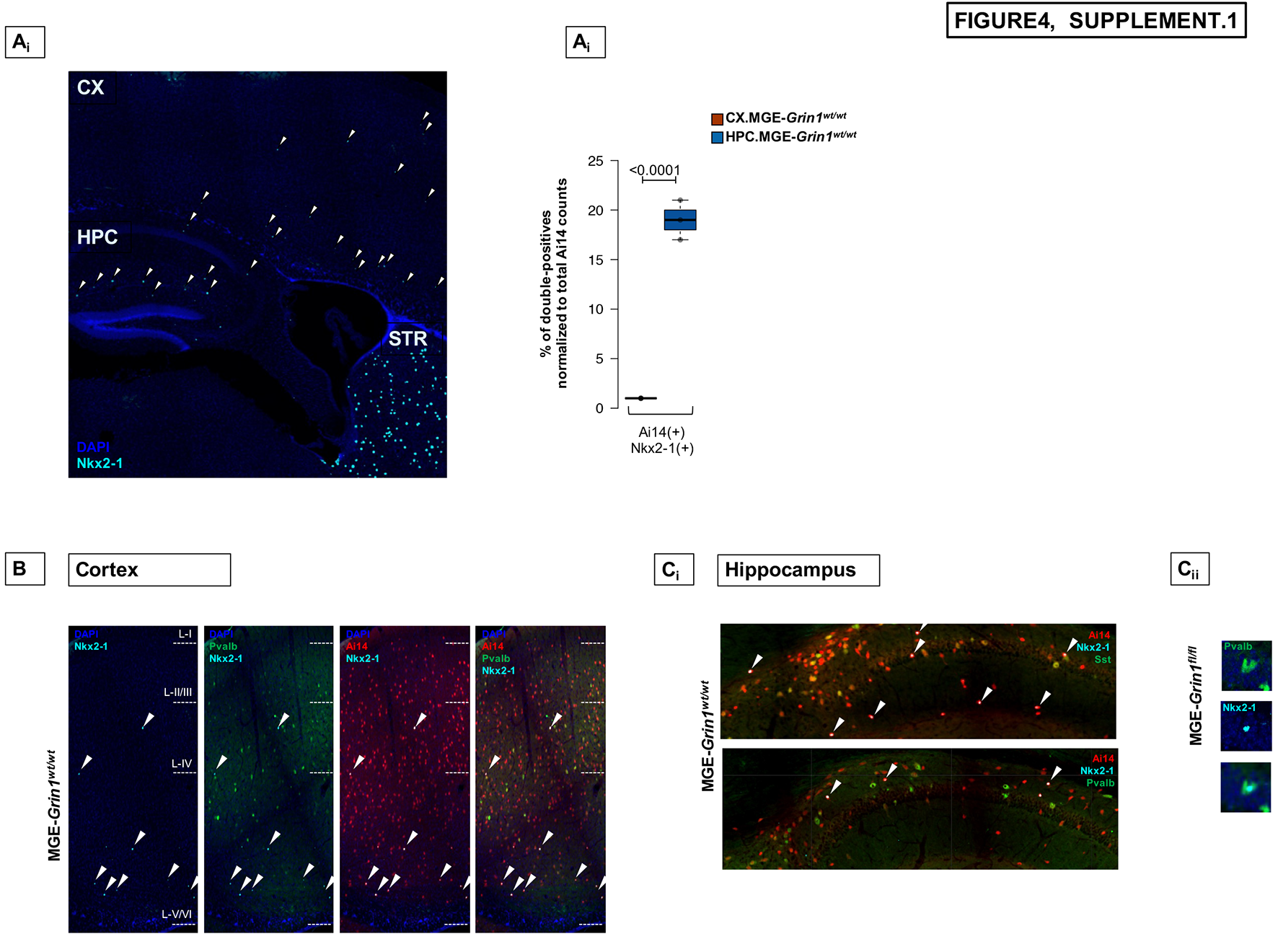

Supplement: Supplementary file 11 [file Image_10.TIF]

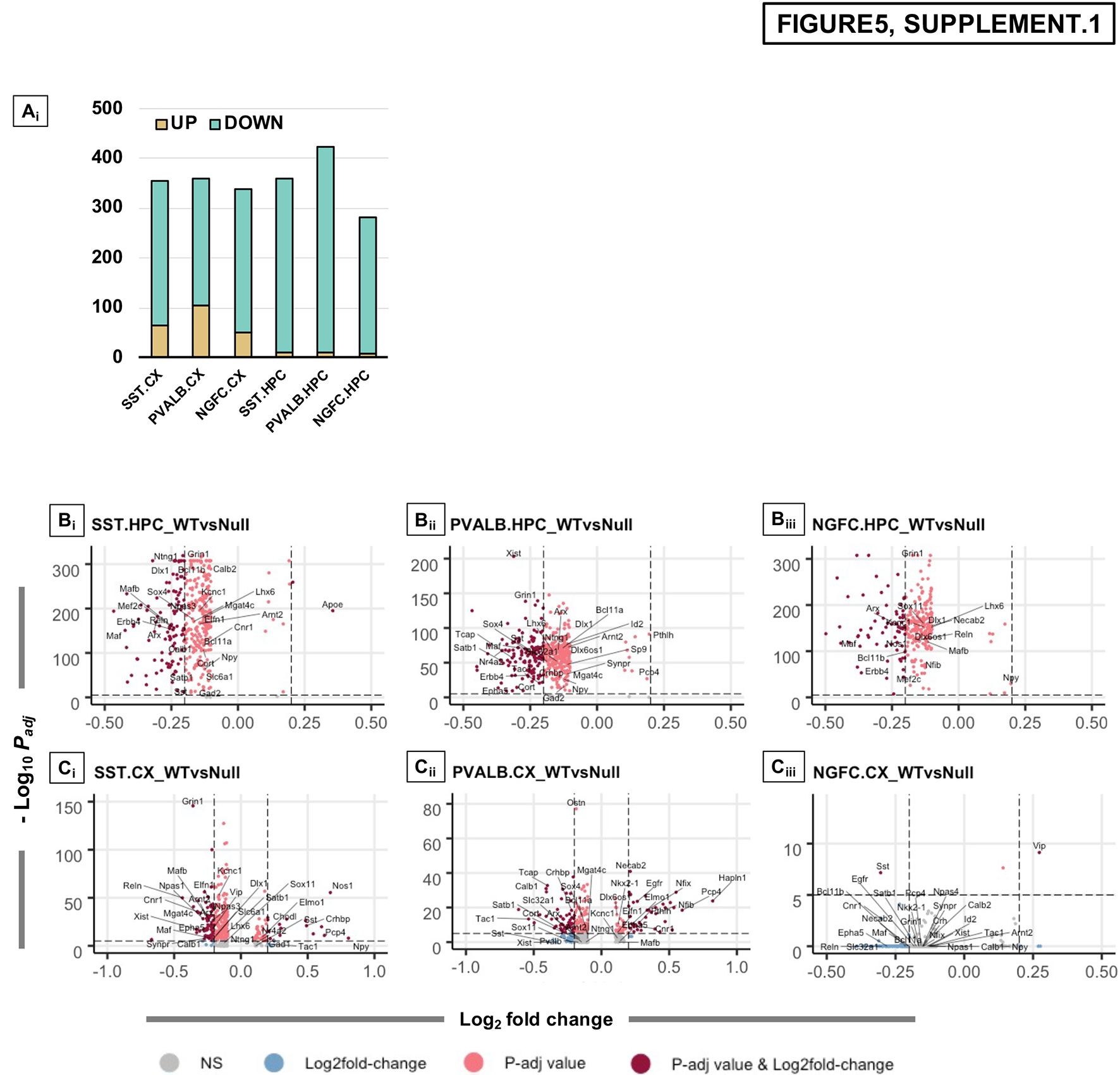

Supplement: Supplementary file 12 [file Image_11.TIF]

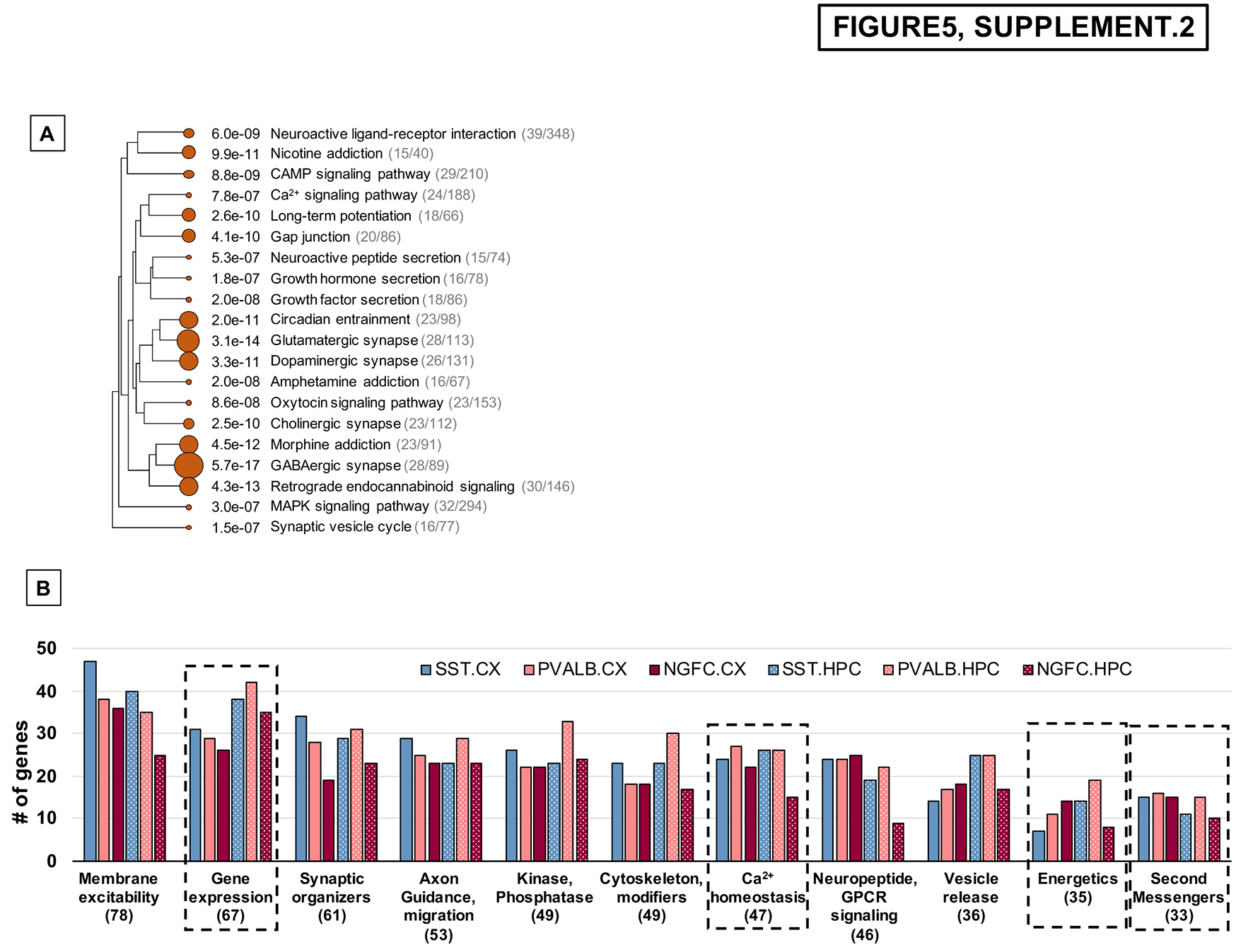

Supplement: Supplementary file 13 [file Image_12.TIF]

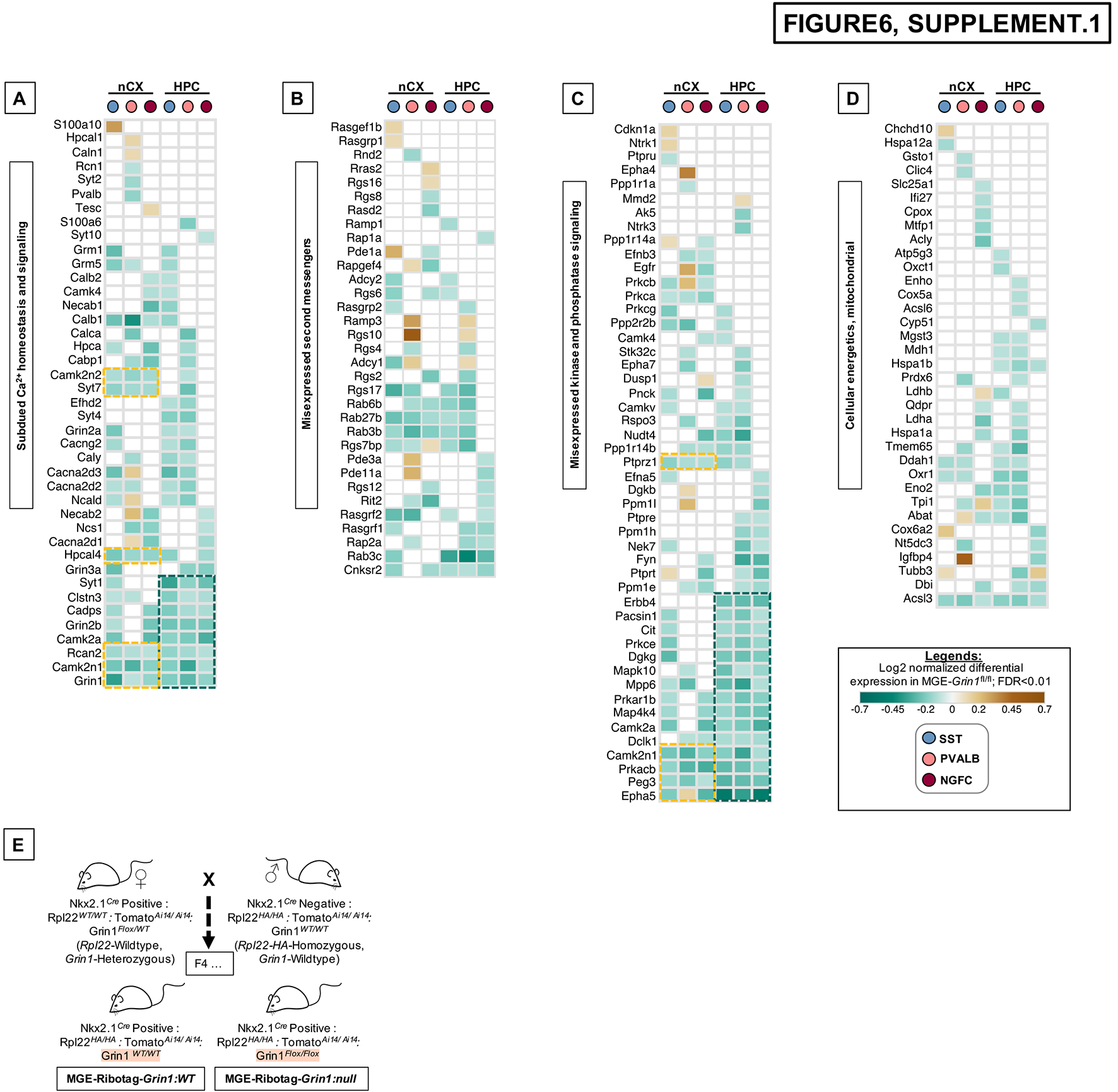

Supplement: Supplementary file 14 [file Image_13.TIF]

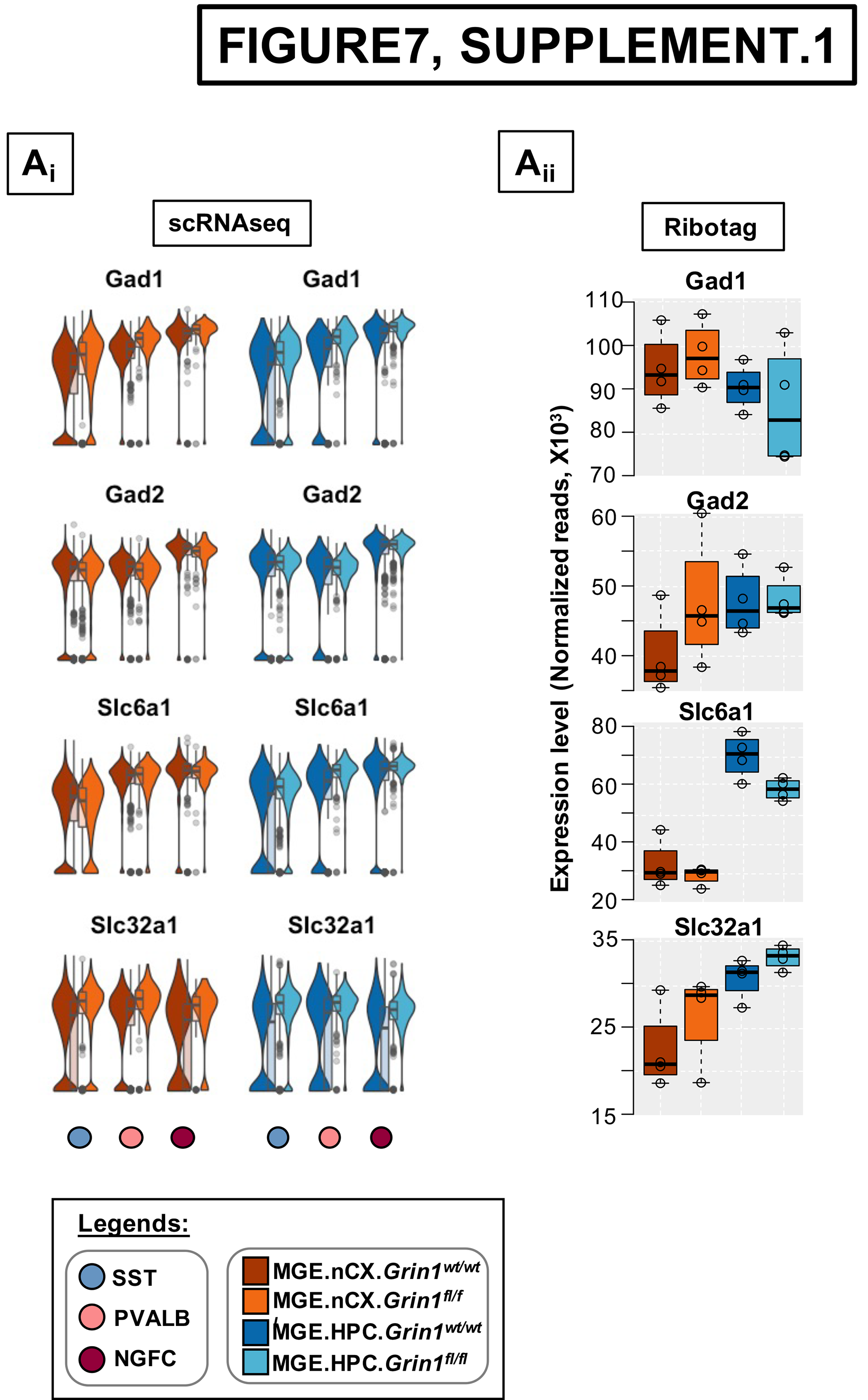

Supplement: Supplementary file 15 [file Image_14.TIF]

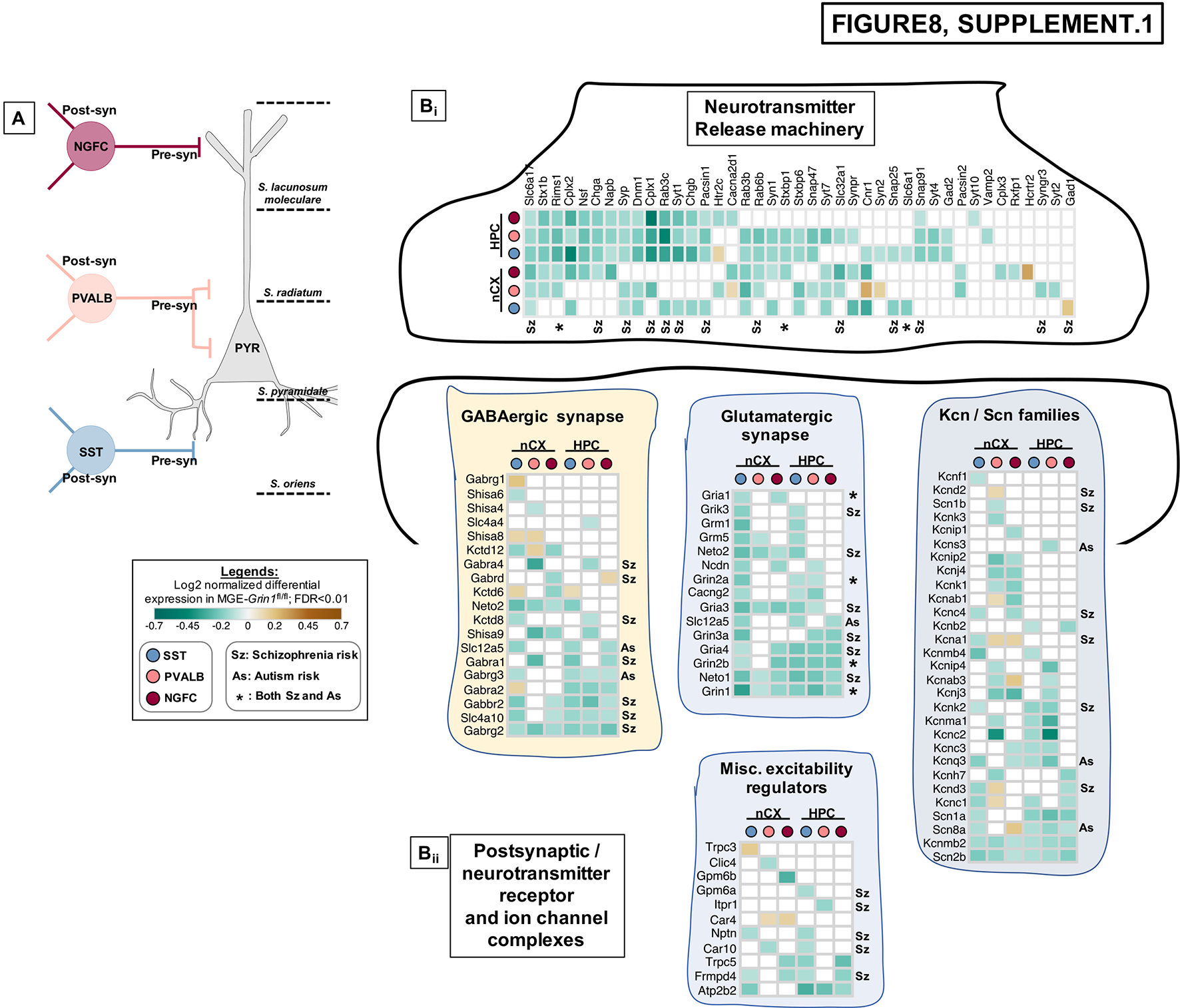

Supplement: Supplementary file 16 [file Image_15.TIF]
